# Coordination Versatility of Thiazolidinone-Based Ligands toward Ag**^+^**: Structural and Photophysical Insights

**DOI:** 10.1021/acsomega.6c00026

**Published:** 2026-04-15

**Authors:** Julio Corredoira-Vázquez, Manuel Saa, Isabel García-Santos, Alfonso Castiñeiras, Matilde Fondo

**Affiliations:** † Departamento de Química Inorgánica, Facultade de Química, 16780 Universidade de Santiago de Compostela, Campus Vida, Santiago de Compostela 15782, Spain; ‡ Institute of Materials (iMATUS), Universidade de Santiago de Compostela, Santiago de Compostela 15782, Spain; § Departamento de Química Inorgánica, Facultade de Farmacia, Universidade de Santiago de Compostela, Campus Vida, Santiago de Compostela 15782, Spain

## Abstract

The reaction of the thiazolidinone-based ligands HAm4DHotaz, Am4Motaz, and Am4Eotaz with AgClO_4_ in a 1:1 molar ratio yields complexes of varying nuclearities depending on the N_thiazolidinone_ R-substituent. The unsubstituted HAm4DHotaz (R = H) affords the coordination polymer {[Ag­(HAm4DHotaz)]­(ClO_4_)}_*n*_ (**1**) whereas Am4Motaz (R = Me) and Am4Eotaz (R = Et) lead to the discrete mononuclear complexes [Ag­(Am4Motaz)_2_]­(ClO_4_) (**2**) and [Ag­(Am4Eotaz)_2_]­(ClO_4_)] (**3**), respectively, as the main products. From the corresponding mother liquors, small amounts of the coordination polymers {[Ag­(Am4Motaz)­(ClO_4_)]}_*n*_ (**4**) and {[Ag_2_(Am4Eotaz)_2_(H_2_O)­(ClO_4_)]­(ClO_4_)·H_2_O}_*n*_ (**5**·H_2_O) were also obtained. In addition, the reaction of AgNO_3_ with Am4Eotaz in 1:1 or 0.5:1 molar ratio leads to [Ag­(Am4Motaz)_2_]­(NO_3_)·H_2_O (**6**·H_2_O), while the mother liquors of the 0.5:1 experiment produced small amounts of the coordination polymer {[Ag_2_(Am4Motaz)_3_]­(NO_3_)_2_·H_2_O}_*n*_ (**7**·H_2_O). X-ray diffraction analyses of all the complexes highlight the remarkable coordination versatility of these ligands, revealing five distinct binding modes, including the unprecedented tridentate μ_2_-κ^2^NN′:κ^1^N″ and μ_2_-κ^1^N:κ^2^N′S bridging motifs, as well as the two rarely reported modes μ_2_-κ^2^NN′:κ^1^S and μ_2_-κ^3^NN′S:κ^1^. In addition, photophysical studies also highlight a pronounced dependence of the emission properties on the N_thiazolidinone_ R-substituent. Notably, although Ag^+^ coordination quenches ligand-centered emission in all cases, [Ag­(Am4Eotaz)_2_]­(ClO_4_)] (**3**) stands out as the only complex that remains emissive.

## Graphical abstract

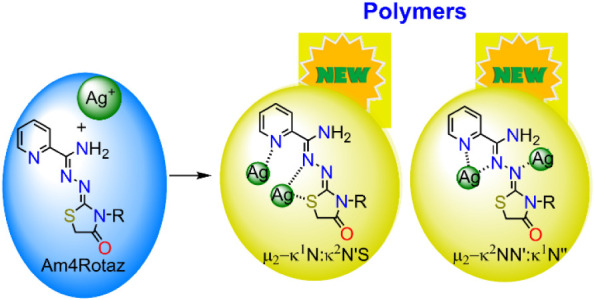

## Introduction

Thiazolidinones constitute a well-established family of organic derivatives that have long attracted attention due to their notable intrinsic pharmacological properties. Although their potential as bioactive pharmacophores is widely recognized, their application in coordination chemistry has been limited by synthetic challenges,^1^ resulting in a limited number of reported metal complexes to date.^2–19^ Consequently, research on thiazolidinone-based ligands and their coordination compounds remains significantly underdeveloped. This scarcity is particularly striking given the synergistic opportunities arising from the combination of the inherent biological activity of the thiazolidinone core with the additional pharmacological potential introduced by metal ions.

Even fewer studies have focused on thiazolidinone Schiff-base-type ligands. Reported examples are largely restricted to derivatives of HTone (2-(2-(1-(pyridin-2-yl)­ethylidene)­hydrazono)­thiazolidin-4-one)^12–14^ or Am4Rotaz (*N*′-(R-4-oxothiazol-idin-2-ylidene)­picolinohydrazonamide).^10,11,16–19^ These systems are particularly noteworthy because they represent the only crystallographically characterized thiazolidinone-based coordination compounds that have also exhibited biological activity.^12,13,16,17^ Nevertheless, the chemistry of this kind of thiazolidinone ligands remains in its early stages, and the relationship between their coordination versatility and potential bioactivity is still poorly understood.

In this context, gaining deeper insight into the coordination behavior of such ligands is essential for advancing their prospective biological applications. Motivated by these considerations, and building upon our expertise in the synthesis of Am4Rotaz ligands and their coordination complexes,^10,16,18,19^ we report a new series of silver­(I) compounds derived from three distinct Am4Rotaz donors. Structural analysis of these compounds highlights the remarkable versatility of this family of ligands, including the identification of two previously unreported coordination modes alongside others that are rarely documented in the literature.

Besides, bioactive luminescent metallodrugs continue to attract growing interest due to their dual potential for therapy and for imaging in living systems. In particular, Ag^+^ complexes containing N-heterocyclic donors have shown luminescence properties that can serve as useful probes for tracking biodistribution.^20,21^ In this context, we also present solution-phase photochemical studies of the ligands and their corresponding silver­(I) complexes with different counterions, as these properties may be relevant for future investigations into their potential biological activity. Notably, the nature of the counterion could exert a pronounced influence on the emission behavior.^22^

## Experimental Section

### Materials and General Methods

All chemical reagents were purchased from commercial sources, and used as received without further purification. Elemental analyses of C, H and N were performed on a Themoscientific Flash Smart analyzer. Electrospray mass spectra (ESI^+^) of the complexes were recorded in methanol solutions in a mass spectrometer Bruker Microtof ESI-TOF. Infrared spectra were registered in the ATR mode on a Bruker IFS-66v spectrophotometer in the range 4000–500 cm^–1^. The ^1^H and ^13^C NMR spectra were recorded on a BRUKER AMX 300 spectrometer. All the IR and NMR raw spectra of the main products **1**–**3** and **6**·H_2_O are shown in the Supporting Information (Figures S1–S3).

The three ligands were previously reported,^10,11,19^ and were synthesized by a microwave-assisted method, as published.^19^

### Synthesis of the Complexes

All the complexes were obtained by a conventional method. In all cases, the solutions obtained and the solids isolated were protected from light with aluminum foil.

The synthesis of the metal complexes by mixing the corresponding Am4Rotaz ligand and the silver salt in 1:1 molar ratio is exemplified by the isolation of {[Ag­(HAm4DHotaz)]­(ClO_4_)}_*n*_ (**1**).

#### {[Ag­(HAm4DHotaz)]­(ClO_4_)}_*n*_ (**1**)

A solution of AgClO_4_ (44 mg, 0.21 mmol) in H_2_O (9 mL) was added to a solution of HAm4DHotaz (50 mg, 0.21 mmol) in MeOH (20 mL), and the mixture was stirred for 15 min. Evaporation of the resulting light-yellow solution, which was protected from light, produced colorless needles of **1** suitable for single X-ray diffraction studies, which were filtered and dried in air. Yield: 32%. Elemental analysis: Found: C, 24.0; H, 1.8; N, 15.5; S, 7.1%. Calc. for C_9_H_9_AgClN_5_O_5_S:C, 24.4; H, 2.0; N, 15.8; S, 7.2%. IR (ν̃/cm^1^): 3461–3312 ν­(NH), 1709 ν­(C=O), 1562–1616 ν­(C=N) + ν­(C=C), 1003 ν­(NN), 1144–1081 ν­(ClO_4_). Mass spectra (ESI^+^, *m*/*z*): 341.96 [Ag­(HAm4DHotaz)]^+^, 579.01 [Ag_2_(HAm4DHotaz)]^+^, 684.9 [Ag_2_(HAm4DHotaz)_2_-H]^+^, 792.8 [Ag_3_(Am4DHotaz)_2_]^+^. ^1^H NMR (DMSOd_6_, δ in ppm, *J* in Hz): 11.72 (1H, s, NH), 8.60 (1H, d, *J* = 4.7, H1), 8.10 (1H, d, *J* = 7.9, H4), 8.00 (1H, dd, *J* = 7.9, 1.3, H3), 7.58 (1H, dd, *J* = 7.4, 1.8 H2), 6.76 (2H, broad, NH_2_), 3.87 (2H, s, CH_2_). ^13^C NMR (DMSO-*d*_6_, δ in ppm): 173.29 (C=O), 157.50 (C7), 153.58 (C6), 148.74 (C5), 149.38 (C1), 138.09 (C3), 126.06 (C2), 121.90 (C4), 33.16 (CH_2_).

#### [Ag­(Am4Motaz)_2_]­(ClO_4_) (**2**)

AgClO_4_: 8.2 mg (0.04 mmol); Am4Motaz: 10 mg (0.04 mmol). Slow evaporation of the solution yielded single crystals of **2** suitable for X-ray diffraction studies. Yield: 52%. Elemental analysis: Found: C, 34.2; H, 3.7; N, 18.1; S, 8.3%. Calc. for C_20_H_22_AgClN_10_O_8_S_2_: C, 34.3; H, 3.9; N, 18.2; S, 8.3%. IR (ν̃/cm^1^): 3440–3315 ν­(NH), 1712 ν­(C=O), 1608–1577 ν­(C=N) + ν­(C=C), 1004 ν­(NN), 1103, 1072 ν­(ClO_4_^–^). Mass spectra (ESI^+^, *m*/*z*): 356.0 [Ag­(Am4Motaz)]^+^, 607.0 [Ag­(Am4Motaz)_2_)]^+^. ^1^H NMR (DMSOd_6_, δ in ppm, *J* in Hz):8.61 (2H, d, *J* = 4.9, H1), 8.14 (2H, d, *J* = 7.9, H4), 8.02 (2H, dd, *J* = 7.9, 1.3, H3), 7.61–7.58 (2H, m, H2), 7.09 (4H, broad, NH_2_), 3.93 (4H, s, CH_2_), 3.23 (6H, s, Me). ^13^C NMR (DMSOd_6_, δ in ppm): 171.51 (C=O), 156.60 (C7), 154.43 (C6), 149.34 (C1), 148.94 (C5), 138.00 (C3), 125.99 (C2), 121.92 (C4), 32.34 (CH_2_), 29.34 (Me).

The same complex is isolated when the reaction is repeated with a 0.5:1 metal salt:ligand ratio. Few single crystals of {[Ag­(Am4Motaz)­(ClO_4_)]}_*n*_ (**4**), suitable for X-ray diffraction studies, were obtained from the mother liquor of this reaction, with a yield lower than 2%.

#### [Ag­(Am4Eotaz)_2_]­(ClO_4_) (**3**)

AgClO_4_: 16 mg (0.076 mmol); Am4Eotaz: 20 mg (0.076 mmol). Single crystals of **3** were obtained by slow evaporation of the solution. Yield: 21%. Elemental analysis: Found: C, 36.3; H, 3.4; N, 19.0; S, 8.5%. Calc. for C_22_H_36_AgClN_10_O_6_S_2_: C, 36.0; H, 3.5; N, 19.1; S, 8.7%. IR (ν̃/cm^1^): 3487–3317 ν­(NH), 1708 ν C=O), 1612–1583 ν­(C=N) + ν­(C=C), 1132, 1081 ν­(ClO_4_^–^). Mass spectra (ESI^+^, *m*/*z*): 372.0 [Ag­(Am4Eotaz)]^+^, 635.1 [Ag­(Am4Eotaz)_2_]^+^. ^1^H NMR (DMSOd_6_, δ in ppm, *J* in Hz): 8.57 (1H, d, *J* = 5.0, H1), 8.13 (2H, d, *J* = 7.9, H4), 8.03 (2H, dd, *J* = 7.9, 1.3, H3), 7.61 (2H, m, H2), 7.16 (4H, broad, NH_2_), 3.91 (4H, s, CH_2_), 3.84 (4H, c, CH_2_, Et), 1.16 (6H, t, CH_3_, Et). ^13^C NMR (DMSO-*d*_6_, δ in ppm): 171.27 (C=O), 155.96 (C7), 154.48 (C6), 149.37 (C1), 148.86 (C5), 138.12 (C3), 126.10 (C2), 122.08 (C4), 32.35 (CH_2_), 37.61 (CH_2_, Et), 12.47 (CH_3_, Et).

Few single crystals of {[Ag_2_(Am4Eotaz)_2_(H_2_O)­(ClO_4_)]­(ClO_4_)·H_2_O}_*n*_ (**5**·H_2_O), suitable for X-ray diffraction studies, precipitated from the mother liquor of this reaction, with a yield lower than 2%.

#### [Ag­(Am4Motaz)_2_]­(NO_3_)·H_2_O (**6**·H_2_O)

AgNO_3_: 14 mg (0.085 mmol); Am4Motaz: 20 mg (0.085 mmol). Evaporation of the solution yielded single crystals of **6**·H_2_O suitable for X-ray diffraction studies. Yield: 60%. Elemental analysis: Found: C, 34.8; H, 3.4; N, 22.3; S, 9.1%. Calc. for C_20_H_24_AgN_11_O4_6_S_2_: C, 35.0; H, 3.5; N, 22.4; S, 9.3%. IR (ν̃/cm^1^): 3573 ν­(OH), 3468–3347 ν­(NH), 1707 ν­(C = O), 1615–1585 ν­(C=N) + ν­(C=C), 999 ν­(NN), 1383 (νNO_3_). Mass spectra (ESI^+^, *m*/*z*): 356.0 [Ag­(Am4Motaz)]^+^, 605.0 [Ag­(Am4Motaz)_2_)]^+^. ^1^H NMR (DMSOd_6_, δ in ppm, *J* in Hz): 8.57 (2H, d, *J* = 4.9, H1), 8.12 (2H, dd, *J* = 7.9, 1.3, H4), 8.04 (2H, dd, *J* = 7.9, 1.3, H3), 7.60–7.57 (2H, m, H2), 7.20 (4H, broad, NH_2_), 3.90 (4H, s, CH_2_), 3.21 (6H, s, Me). ^13^C NMR (DMSO-*d*_6_, δ in ppm): 171.46 (C=O), 156.60 (C7), 154.58 (C6), 149.43 (C1), 148.84 (C5), 138.11 (C3), 126.08 (C2), 122.06 (C4), 32.37 (CH_2_), 29.34 (Me).

The same complex is isolated when the reaction is repeated with a 0.5:1 metal salt:ligand ratio. In addition, few single crystals of {[Ag_2_(Am4Motaz)_3_]­(NO_3_)_2_·H_2_O}_*n*_ (**7**·H_2_O) were obtained from the mother liquor of this latter reaction, with a yield lower than 2%.

### Single X-ray Diffraction Studies

Diffraction data for {[Ag­(HAm4DHotaz)]­(ClO_4_)}_*n*_ (**1**), [Ag­(Am4Motaz)_2_]­(ClO_4_) (**2**), [Ag­(Am4Eotaz)_2_]­(ClO_4_)] (**3**), {[Ag­(Am4Motaz)­(ClO_4_)]}_*n*_ (**4**), {[Ag_2_(Am4Eotaz)_2_(H_2_O)­(ClO_4_)]­(ClO_4_)·H_2_O}_*n*_ (**5**·H_2_O), [Ag­(Am4Motaz)_2_]­(NO_3_)·H_2_O (**6**·H_2_O) and {[Ag_2_(Am4Motaz)_3_]­(NO_3_)_2_·H_2_O}_*n*_ (**7**·H_2_O) were recorded at 100.0(2) K on a Bruker X8 KappaAPEXII diffractometer. Graphite monochromated MoK­(α) radiation (λ = 0.71073 Å) was used throughout. The data were processed with APEX2 ^23^ and corrected for absorption using SADABS.^24^ The structures were solved by direct methods^25^ and refined by full-matrix least-squares techniques against *F* ^2^.^26^ Positional and anisotropic atomic displacement parameters were refined for all non-hydrogen atoms. Hydrogen atoms were located in difference maps and included as fixed contributions riding on attached atoms with isotropic thermal parameters 1.2/1.5 times those of their carrier atoms. Molecular graphics were generated with DIAMOND.^27^ Details on experimental and refinement results are summarized in Table S1. CCDC 2490314–2490315 and 2490317–2490321 contain the supplementary crystallographic data. These data can be obtained free of charge via https://www.ccdc.cam.ac.uk/structures or from the Cambridge Crystallographic Data Center, 12 Union Road, Cambridge CB2 1EZ, UK; fax: (+44)-1223-336-033; or e-mail: deposit@ccdc.cam.ac.uk.

### Powder X-ray Diffraction Studies

The powder diffractograms for **1**–**3** and **6**·H_2_O were recorded in a Philips diffractometer with a control unity type “PW1710”, a vertical goniometer type “PW1820/00” and a generator type “Enraf Nonius FR590”, operating at 40 kV and 30 mA, using monochromated CuKα (λ = 1.5418 Å) radiation. A scan was performed in the range 2 < 2θ < 40° with t = 3 s and Δ2θ = 0.02°. Le Bail refinement was obtained with the aid of HighScore Plus Version 3.0d.

### Photophysical Studies

The UV–vis and fluorescence emission spectra for the three ligands and metal complexes **1**, **2**, **3** and **6**·H_2_O in methanol solution (concentrations 10^–4^ M, 5 × 10^–5^ M, 10^–5^ M, 5 × 10^–6^ M) were recorded on a JASCO V-730 spectrophotometer and on a SHIMADZU RF-6000 spectrofluorometer, respectively. All the measurements were taken at 298 K.

## Results and Discussion

### Synthesis and Spectroscopic Studies

Reaction of related HAm4DHotaz, Am4Motaz and Am4Eotaz^16,17,19^ with AgClO_4_ was studied, as summarized in Scheme 1. Thus, reaction of this salt in 1:1 molar ratio with HAm4DHotaz leads to the coordination polymer {[Ag­(HAm4DHotaz)]­(ClO_4_)}_*n*_ (**1**), while with Am4Motaz or Am4Eotaz yield the mononuclear complexes [Ag­(Am4Motaz)_2_]­(ClO_4_) (**2**) and [Ag­(Am4Eotaz)_2_]­(ClO_4_)] (**3**) as the main products. The same products **2** and **3** can be obtained by mixing the salt and ligand in 0.5:1 molar ratio.

**Scheme 1:**
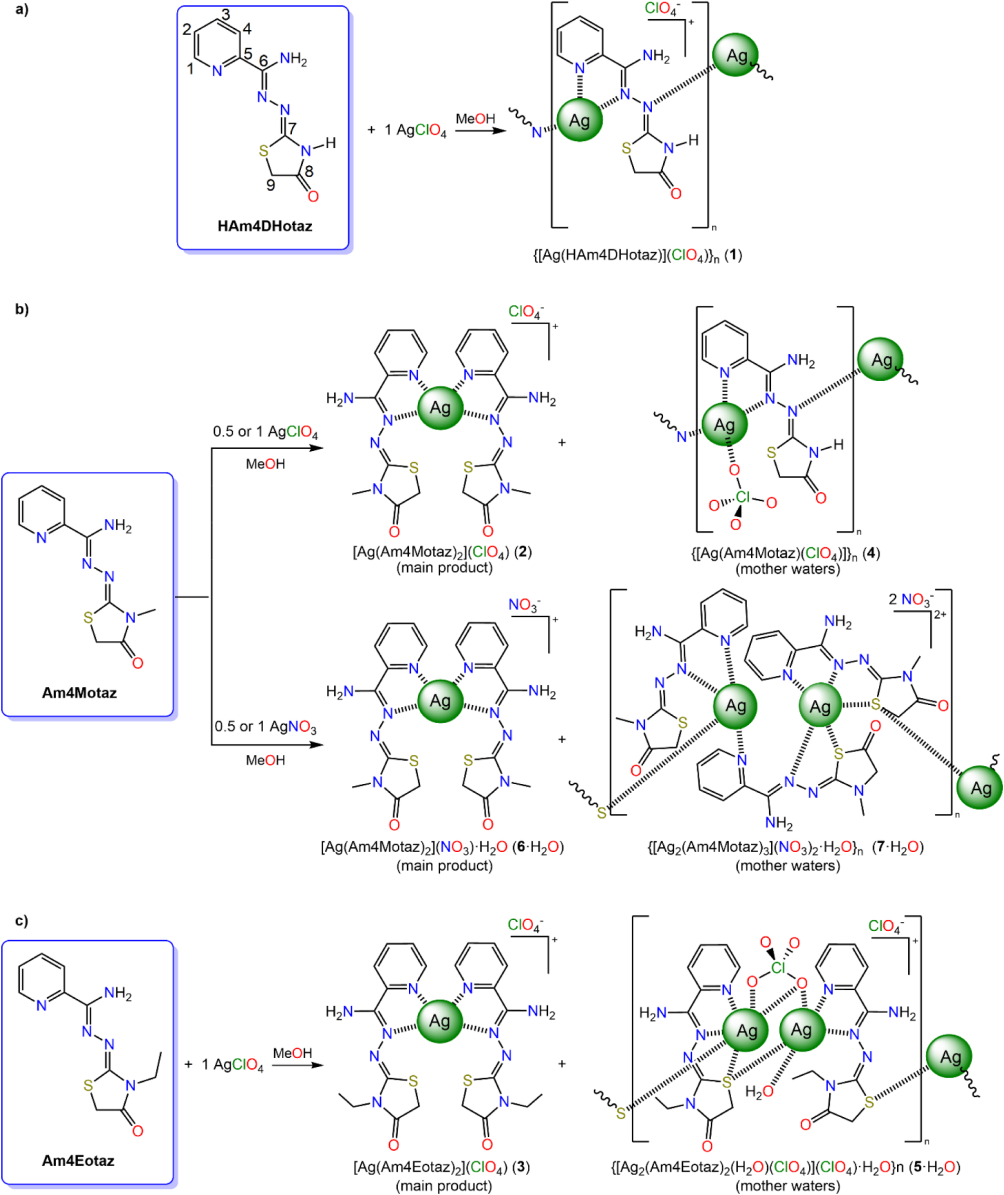
Ligands Employed in this Work (a, b, c), with Numbering for NMR Spectroscopy in Ham4DHotaz (a), and Reaction Schemes for Isolation of Silver Complexes of HAm4DHotaz (a), Am4Motaz (b) and Am4Eotaz (c). The water Solvate is Omitted for Clarity

However, the mother liquors of these latter reactions also produce a small quantity of the coordination polymers {[Ag­(Am4Motaz)­(ClO_4_)]}_*n*_ (**4**) and {[Ag_2_(Am4Eotaz)_2_(H_2_O)­(ClO_4_)]­(ClO_4_)·H_2_O}_*n*_ (**5**·H_2_O), which could be identified by X-ray diffraction studies (Schemes 1b and c). These findings suggest that the R substituents on the N atom of the thiazolidinone ring play a decisive role in determining the nuclearity of the isolated complexes. In particular, as the steric bulk of the substituent increases, the formation of coordination polymers as main products seems to be progressively disfavored, giving rise instead to monomeric species. In addition, the reaction of AgNO_3_ with these ligands in 1:1 or 0.5:1 molar ratios leads to [Ag­(L)_2_]­(NO_3_)·*n*H_2_O complexes, as previously described for HAm4DHotaz and Am4Eotaz,^19^ and illustrated in Scheme 1b for Am4Motaz. In this latter case, in addition to the main product [Ag­(Am4Motaz)_2_]­(NO_3_)·H_2_O (6·H_2_O), the mother waters of the 0.5:1 molar ratio produced a small quantity of single crystals of the coordination polymer {[Ag_2_(Am4Motaz)_3_]­(NO_3_)_2_·H_2_O}_*n*_ (7·H_2_O).

Accordingly, the present findings indicate that, for salts bearing non-basic anions, the counterion exerts no significant influence on the nature of the isolated main products.

All the complexes were characterized by single X-ray diffraction studies. In addition, main complexes **1**, **2**, **3** and **6**·H_2_O were fully characterized by elemental analysis, mass spectrometry, IR and ^1^H NMR spectroscopy. In contrast, the limited amounts of the byproducts **4**, **5**·H_2_O and **7**·H_2_O prevented their complete characterization.

For mononuclear [Ag­(L)_2_]­X compounds (**2**, **3**, and **6**·H_2_O), mass spectrometry allows identifying [Ag­(L)_2_]^+^ fragments, in agreement with the 0.5:1 Ag:L stoichiometry. Nevertheless, for the coordination polymer **1**, peaks corresponding to the polynuclear species [Ag_2_(HAm4DHotaz)­(Am4DHotaz)]^+^ and [Ag_3_(Am4DHotaz)_2_]^+^ could be also identified. Thus, in this case the ESI^+^ mass spectrometry seems a useful technique to distinguish between mononuclear and complexes of higher nuclearity.

The IR spectra of the main products (Figure S1) exhibit bands corresponding to ν­(NH) vibrations in the 3312-3490 cm^–1^ range,^28^ which are shifted to lower frequencies compared to the free ligands.^10,11^ Additionally, the spectrum of **6**·H_2_O displays a broad band around 3570 cm^–1^, assigned to the ν­(OH) vibration of H_2_O. Bands associated with the ν­(C=N) and ν­(C=C) vibrations appear in the 1562–1617 cm^–1^ range. These bands are shifted to lower frequencies relative to the free ligands, consistent with the coordination of the imine N atom to Ag^+^. The strong band around 1700 cm^–1^ in all the spectra corresponds to the ν­(C=O) vibration. Its frequency, similar to that of the free ligand, suggests that this group remains uncoordinated.

Moreover, two or three intense bands in the range 1144–1072 cm^–1^ in the spectra of {[Ag­(HAm4DHotaz)]­(ClO_4_)}_*n*_ (**1**), [Ag­(Am4Motaz)_2_]­(ClO_4_) (**2**) and [Ag­(Am4Eotaz)_2_]­(ClO_4_) (**3**) are consistent with the presence of perchlorate groups in the complexes.^29^ The splitting of the Cl–O vibrations could be attributed to the coordination of the perchlorate to the metal, which would break its symmetry. However, it should be noted that perchlorate can act as a counterion involved in hydrogen bonding, which likewise disrupts its perfect Td symmetry.

In the case of [Ag­(Am4Motaz)_2_]­(NO_3_)·H_2_O (**6**·H_2_O), a band at 1383 cm^–1^ in the IR spectra supports the presence of NO_3_^–^ in the complex.^18^

The ^1^H NMR spectra of **1**, **2**, **3** and **6**·H_2_O (Figure S2) display a single set of signals, consistent with the absence of side products. Key features include:

1. A signal at 11.7 ppm in the spectrum of {[Ag­(HAm4DHotaz)]­(ClO_4_)}_*n*_, assigned to the NH proton of the thiazolidinone ring, supporting the neutral character of the ligand.
2. NH_2_ signals at 6.76 ppm for the HAm4DHotaz complex **1** and at *ca*. 7.2 ppm for Am4Motaz and Am4Eotaz-derived complexes **2**, **3** and **6**·H_2_O, notably more deshielded, and shifted downfield relative to the free ligands in all cases. This is consistent with charge delocalization and coordination effects upon conjugation.
3. H^+^_Py_ peaks between 7–9 ppm, and CH_2_ signals of the thiazolidinone ring at 3.8–4.0 ppm, with minimal shift upon ligand coordination.^10,11^

4. Two peaks at 3.84 and 1.16 ppm for the Am4Eotaz complex **3**, or a peak at *ca*. 3.2 ppm for Am4Motaz compounds **2** and **6**·H_2_O, all of them corresponding to the aliphatic substituents on the thiazolidinone ring.

Furthermore, none of the spectra show signs of decomposition, confirming the short-term stability of these complexes in DMSO*d*_6_ under light exposure (*ca*. 3 h).

The ^13^C NMR spectra (Figure S3) further support the conclusions drawn from the ^1^H NMR data.

### X-ray Diffraction Studies

The crystal structures of the mononuclear complexes [Ag­(Am4Motaz)_2_]­(ClO_4_) (**2**), [Ag­(Am4Eotaz)_2_]­(ClO_4_)] (**3**) and [Ag­(Am4Motaz)_2_]­(NO_3_)·H_2_O (**6**·H_2_O), and those of the coordination polymers {[Ag­(HAm4DHotaz)]­(ClO_4_)}_*n*_ (**1**), {[Ag­(Am4Motaz)­(ClO_4_)]}_*n*_ (**4**), {[Ag_2_(Am4Eotaz)_2_(H_2_O)­(ClO_4_)]­(ClO_4_)·H_2_O}_*n*_ (**5**·H_2_O) and {[Ag_2_(Am4Motaz)_3_]­(NO_3_)_2_·H_2_O}_*n*_ (**7**·H_2_O) could be solved, as detailed in the experimental section. The experimental data for their Xray diffraction analyses are summarized in Table S1.

### Mononuclear Complexes

The structures of the three mononuclear complexes **2**, **3** and **6**·H_2_O are very similar, and they will be discussed together. Their structures are shown in Figures 1 and S4, and their main bond distances and angles are recorded in Table S2.

**Figure 1:**
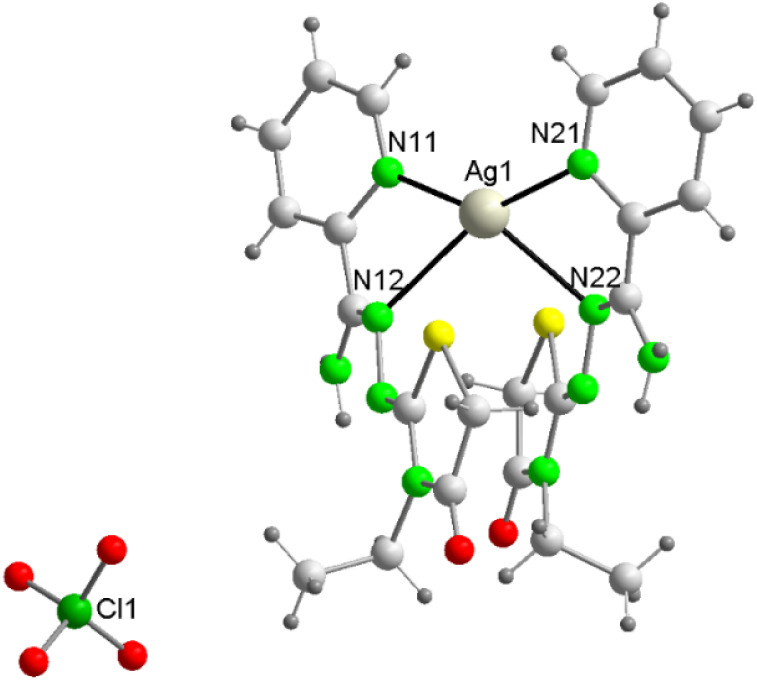
Molecular structure for [Ag­(Am4Eotaz)_2_]­(ClO_4_) (**3**).

These complexes are very similar to the previously reported [Ag­(HAm4DHotaz)_2_]­(NO_3_) and [Ag­(Am4Eotaz)_2_]­(NO_3_).^19^ Thus, all of them are ionic, containing [Ag­(L)_2_]^+^ cations and perchlorate or nitrate counteranions. In addition, water as solvate is present in the unit cell of **6**·H_2_O. As in the related complexes, in the [Ag­(L)_2_]^+^ cations, the ligands act in their more common coordination mode, as neutral bidentate chelate donors, using their pyridine and imine N atoms (κ^2^NN’ coordination mode in Scheme 2a), with the thiazolidinone ring remaining uncoordinated.^10,11,16,17,19^

**Scheme 2:**
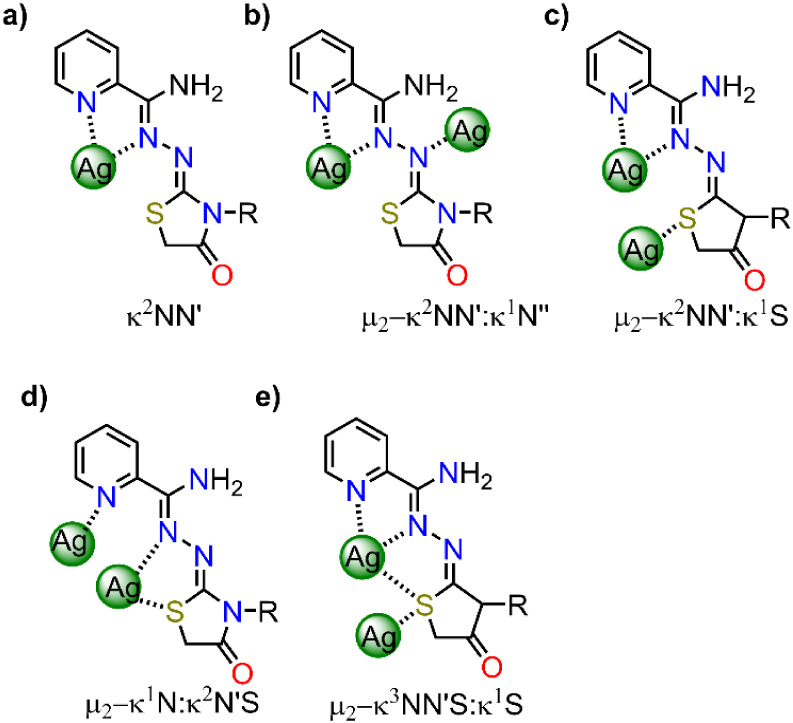
Coordination Modes for Am4Rotaz Ligands in Complexes **1**–**7**·H_2_O

Accordingly, this leads to an *AgN*_4_ core, with Ag^+^ showing a distorted tetrahedral geometry. In all complexes, all the distances and angles agree with the expected ones,^18,19^ and do not merit further consideration.

For the three compounds, hydrogen bonds are established between the carbonyl oxygen atom of the thiazolidinone residue of one ligand (O1 or O2) and the NH_2_ group of a neighboring complex (Table S3). Besides, the perchlorate or nitrate counterions are held in the network by hydrogen bonds with the NH_2_ moieties of the ligands, or in the case of **6**·H_2_O, with the water solvate, which break their symmetry. It is remarkable that the presence of the perchlorate or nitrate counterion seems to have a profound impact on the packing, as the shortest Ag···Ag distance in the perchlorate complex of Am4Motaz **2** is 12.227(4) Å, while for the nitrate complex **6**·H_2_O is 6.9423(4) Å.

### Coordination Polymers

#### {[Ag­(HAm4DHotaz)]­(ClO_4_)}*_n_* (**1**) and {[Ag­(Am4Motaz)­(ClO_4_)]}*_n_* (**4**)

Both polymers are similar, and they will be discussed together. Views of their structures are shown in Figures 2 and 3, and main bond distances and angles in Table S4.

**Figure 2:**
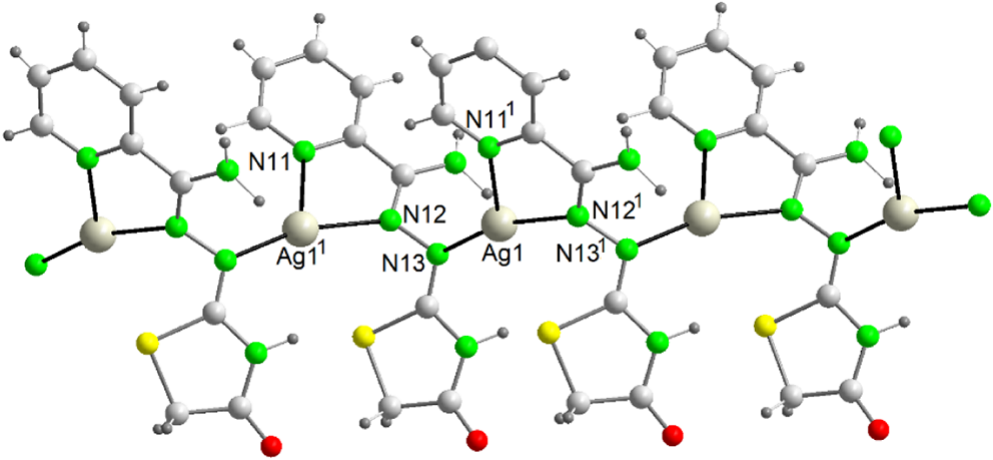
A view of the polymeric cation {[Ag­(HAm4DHotaz)]^+^}_*n*_ in {[Ag­(HAm4DHotaz)]­(ClO_4_)}_*n*_ (**1**) Symmetry code: ^1^ x, −y + 1/2, z – 1/2.

**Figure 3:**
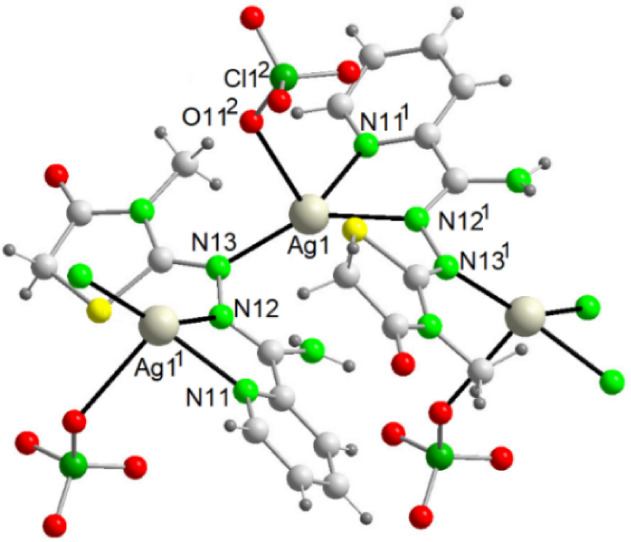
A fragment of the polymeric chain {[Ag­(Am4Motaz)­(ClO_4_)]}_n_ (**4**). Symmetry codes: ^1^ −x, y – 1/2, −z + 1/2; ^2^ −x, −y + 1, −z.

Both complexes are one-dimensional coordination polymers built from mononuclear units, albeit of different nature. In {[Ag­(HAm4DHotaz)]­(ClO_4_)}_*n*_, the cationic polymer (Figure 2) arises from the linkage of [AgL]^+^ cations, with ClO_4_^–^ counteranions balancing the charge (minimum Ag···O distance = 3.1839(3) Å), whereas the {[Ag­(Am4Motaz)­(ClO_4_)]}_*n*_ polymeric chain (Figure 3) is based on [Ag­(Am4Motaz)­(ClO_4_)] molecules, owing to the monodentate coordination (κ^1^-O) of the perchlorate (distance Ag1–O11^1^ = 2.740(2) Å). Despite this difference, both compounds share many common features. Thus, in both cases the Ag^+^ centers are coordinated to a neutral bidentate chelating ligand via the imine (N12) and pyridine (N11) nitrogen atoms, and to the N13 atom of the carbohydrazone residue from an adjacent [AgL]^+^ unit, giving rise to a μ_2_-κN:κ^2^N′N″ tridentate bridging mode (Scheme 2b). Remarkably, this coordination mode has not previously been reported for this type of ligand. Additionally, weak secondary interactions with the thiazolidinone sulfur atoms (distance Ag···S = 3.2 Å) are also observed.

Thus, in {[Ag­(HAm4DHotaz)]­(ClO_4_)}_*n*_, the Ag^+^ centers adopt a *N*_3_ coordination environment. Calculations with the SHAPE program^30^ indicate that the geometry is distorted mer-trivacant octahedron or T-shape (Table S5, N–Ag–N angles = 70.7–163°). In {[Ag­(Am4Motaz)­(ClO_4_)]} the Ag^+^ centers are tetracoordinated in a highly distorted *N*_3_ *O* seesaw environment (Table S5), the O atom coming from a ClO_4_^–^ ligand.

The average Ag–N distances of these compounds (2.271 and 2.305 Å, respectively) are consistent with those reported for *AgN*_3_ ^31^ and *AgN*_3_ *O* ^32^ systems. The relatively long Ag–O distance in **4** is likewise comparable to other AgOClO_3_ bonds.^33^ The Ag···Ag separation in **1** (4.756 Å) and **4** (5.061 Å) remains well above the sum of the van der Waals radii (3.44 Å), thus ruling out argentophilic interactions.

A noteworthy structural difference between the two complexes is that in **1** the thiazolidinone ring of HAm4DHotaz consistently adopts the same orientation, whereas in **4** the rings alternate between upward and downward orientations.

Finally, it is remarkable that the ClO_4_^–^ ligands in **4** connect the 1D chains via N–H···O hydrogen bonds (Table S6), giving rise to a 2D sheet, while in **1** are merely interacting with NH_2_ groups but do not contribute to chain expansion.

#### {[Ag_2_(Am4Eotaz)_2_(H_2_O)­(ClO_4_)]­(ClO_4_)*·*H_2_O}_*n*_ (**5** *·*H_2_O)

This structure is built on the dinuclear cationic block [Ag_2_(Am4Eotaz)_2_(H_2_O)­(ClO_4_)]^+^, with a perchlorate anion and one crystallization water molecule per cation (Figure 4). Its main bond distances and angles are summarized in Table S7.

**Figure 4:**
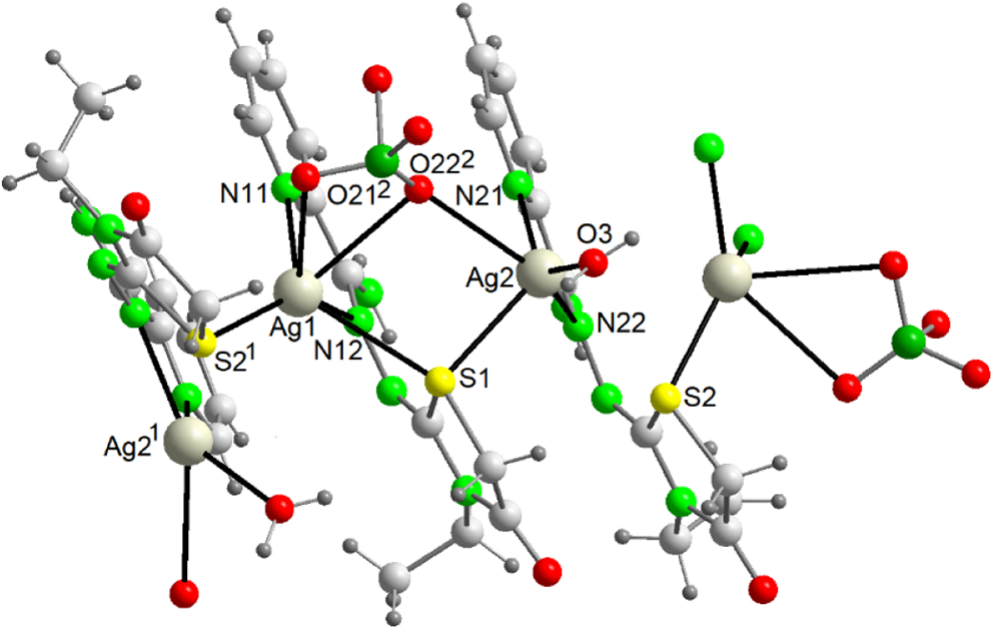
View of a fragment of the polymeric cationic chain [Ag_2_(Am4Eotaz)_2_(H_2_O)­(ClO_4_)]^+^ in {[Ag_2_(Am4Eotaz)_2_(H_2_O)­(ClO_4_)]­(ClO_4_)·H_2_O}_*n*_. Symmetry codes: ^1^ x – 1/2, −y + 3/2, z; ^2^ x, y + 1, z.

In this compound, the two crystallographically independent silver atoms exhibit different coordination environments. Ag1 is bonded to the pyridine (N11) and imine (N12) nitrogen atoms, as well as to the sulfur (S1) atom of the same neutral Am4Eotaz ligand. Additionally, Ag1 is also linked to a second sulfur atom (S2^1^) from a ligand of a neighboring dinuclear block, and it completes its coordination sphere with a perchlorate group coordinated to this atom as bidentate chelate. The Ag–O_perchlorate_ distances of *ca*. 2.9 Å may seem long to be considered true dative covalent bonds, but they are not unusual, and precedents in the literature report Ag–O distances greater than 2.9 Å for other perchlorate ligands.^34^ Therefore, the geometry around Ag1 is a distorted octahedron, with *trans* and *cis* angles ranging from 64.83(11)° to 142.52(11)° (Table S7).

Ag2 also coordinates to an Am4Eotaz ligand through the pyridine (N21) and imine nitrogen (N22) atoms. However, in this case, the Ag2···S2 distance of 3.078(1) Å exceeds the commonly accepted maximum distance of 3.01 Å for Ag–S bonds, and thus cannot be considered a true dative covalent bond. This S2 atom is used to bind one Ag atom of a neighboring dinuclear block, so ligand 2 acts as a μ_2_-κ^2^NN′:κ^1^S bridge (Scheme 2c). The coordination sphere of Ag2 is completed by a sulfur atom from ligand 1 (S1), which now functions as a μ_2_-κ^3^NN′S:κ^1^S bridge (Scheme 2e), a water molecule, and an oxygen atom from the ClO_4_^–^ ligand coordinated to Ag1 as a bidentate chelate. Consequently, the perchlorate ligand adopts a μ_2_-κ^2^OO′:κ^1^O coordination mode. This coordination motif is not without precedent but there are a few examples of Ag^+^ complexes where the poorly coordinating perchlorate anion adopts this mode.^33–37^ The two bridging coordination modes of the Am4Eotaz ligands are also uncommon, as they have only been reported once before.^19^ As a result of the described features, Ag2 is in an *N*_2_ *O*_2_ *S* environment with a distorted square-pyramidal geometry (Addison parameter τ = 0.128).^38^

The dinuclear cations show a double O,S bridge between Ag centers, which gives rise to an *Ag*_2_ *OS* core. Besides, as described, the contiguous Ag atoms of different blocks are bridged by an *NNCS* moiety (μ_2_-κ^2^NN′:κ^1^S bridge). The different intra- and interdinuclear bridges results in two types of Ag···Ag distances within the 1D chain: a longer distance of 4.2586(5) Å within the dinuclear cation [Ag_2_(Am4Eotaz)_2_(H_2_O)­(ClO_4_)]^+^, and shorter distances of 3.35 Å between dinuclear units. The former distance excludes any significant metal–metal interaction within the cation, whereas the intercation Ag···Ag distance is shorter than the sum of the van der Waals radii, though greater than 3.0 Å, indicating weak argentophilic interactions.

All Ag–N, Ag–O and Ag–S distances fall within the ranges found for complexes with the same or similar ligands, and will not be further commented on.^18,19^

Finally, in the crystal, H-bonds are present between neighboring chains (Table S8), involving NH_2_ groups and the thiazolidinone oxygen atoms, forming a 2D supramolecular association. Additionally, the perchlorate anion connects the layers via hydrogen bonding, giving rise to a 3D structure.

#### {[Ag_2_(Am4Motaz)_3_]­(NO_3_)_2_ *·*H_2_O}_*n*_ (**7** *·*H_2_O)

This is a one-dimensional ionic polymer based on the dinuclear [Ag_2_(Am4Motaz)_3_]^2+^ cation. Moreover, the unit cell contains two nitrate anions and one crystallization water molecule per cation. Images of the different parts of polymeric chain are shown in Figure 5, and main bond distances and angles are summarized in Table S9.

**Figure 5:**
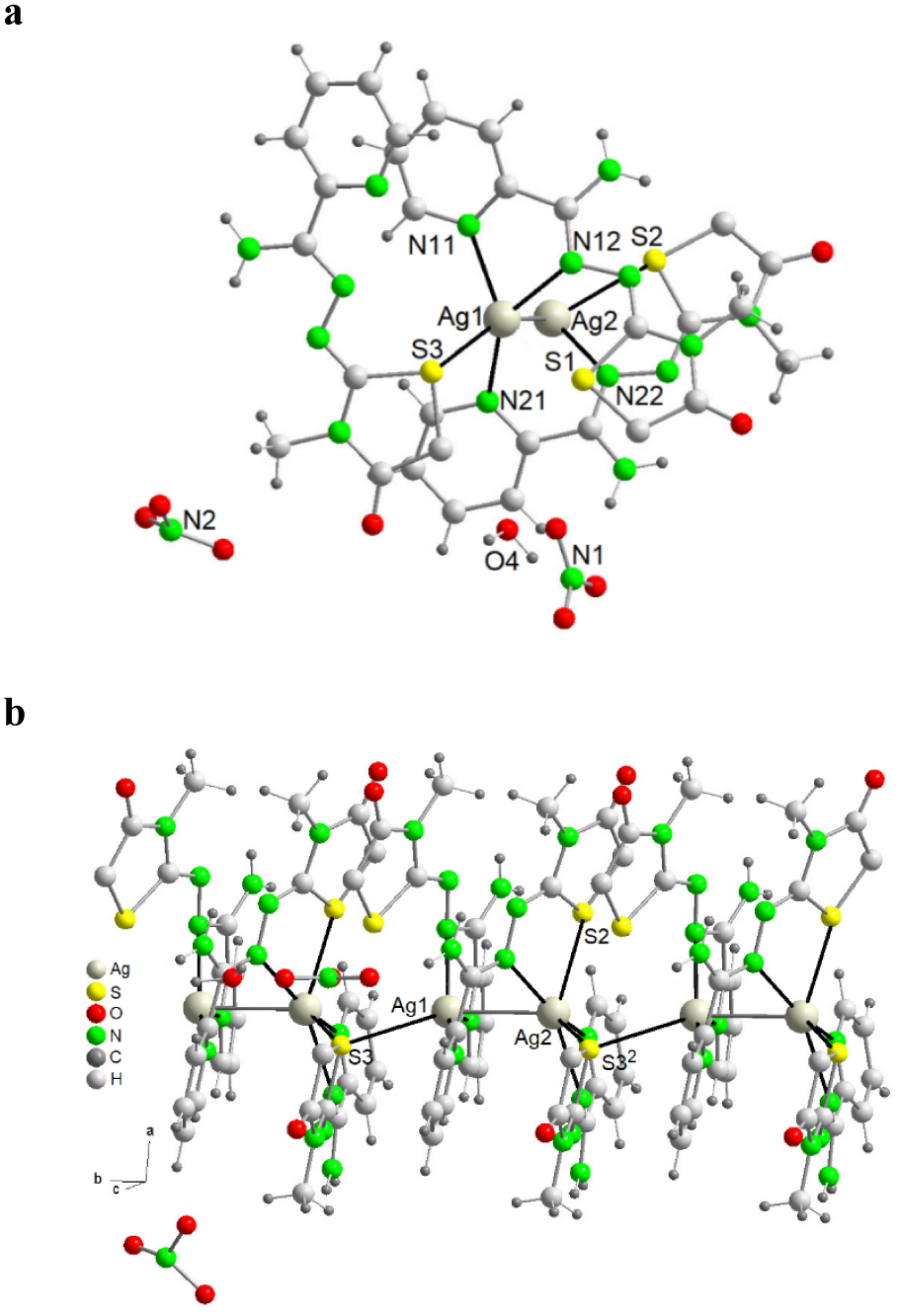
View of the structure of {[Ag_2_(Am4Motaz)_3_]­(NO_3_)_2_·H_2_O}_*n*_ (**7**·H_2_O), showing: a) the asymmetric unit and coordination around the silver ions; b) the polymer chain along the crystallographic *b* axis. Symmetry code: ^2^ x, y – 1, z.

The dinuclear unit contains two crystallographically independent silver atoms (Ag1 and Ag2) with different coordination environments, and three neutral ligands. Ag1 is bonded to two nitrogen atoms (N11, N12) of one ligand, a pyridine-N atom from a second ligand (N21) and a sulfur atom (S3) from a third donor. Furthermore, Ag2 is located at 2.9401(6) Å. This distance is less than 3 Å, but there is an *NCCN* (N21C25C26N22) bridge between Ag1 and Ag2. Accordingly, this interaction is supported by other ligands and, therefore, it cannot be considered a real Ag–Ag bond, but a strong argentophilic interaction. With this, Ag1 is in a tetracoordinated *N*_3_ *S* environment, and, according to SHAPE, its geometry can be described as a distorted tetrahedron (Table S10). The distortion is reflected in the wide range of angles, varying from 71.99(14) to 144.66(14)°.

The Ag2 metal center is bonded to one nitrogen (N22) and a sulfur atom (S2) from the second ligand of the asymmetric unit, and to the pyridine and imine nitrogen (N31^2^ and N32^2^; ^2^ = x, y – 1, z) and the thiazolidinone sulfur atom (S3^2^) from a third ligand of a different dinuclear unit. This latter Ag–S bond is quite long (distance Ag2–S3^2^ of 3.0072(12) Å) but it is in the limit of the commonly accepted maximum distance for AgS bonds (3.01 Å). Thus, all this leads to a *N*_3_ *S*_2_ environment, and SHAPE measurements indicate that the geometry is closer to a pentagon (Table S10), with angles ranging between 63.77(11)° and 151.14(3)°. These angles also show the distortion from the ideal geometry (ideal angles of 72° and 144°).

From the above, it can be inferred that the polymer {Ag_2_(Am4Motaz)_3_(NO_3_)_2_·H_2_O}_*n*_ exhibits three neutral ligands with different coordination modes: thus, ligand 1 acts as a terminal κ^2^NN′ donor (Scheme 2a), ligand 2 as an intradinuclear μ_2_-κ^1^N:κ^2^N′S bridge (Scheme 2d), joining Ag1 and Ag2, while ligand 3 joins the dinuclear blocks in a μ_2_-κ^2^NN′:κ^1^S fashion (Scheme 2e). It is worth noting that the μ_2_-κ^1^N:κ^2^N′S bridging mode has not also been reported before.

As a result of the described features, a single S-bridge links adjacent Ag atoms from different dinuclear units, resulting in an Ag···Ag interdinuclear distance of 4.5612(6) Å, thus ruling out argentophilic interactions between the dinuclear blocks.

Finally, each polymeric chain interacts with adjacent chains through strong NH···O_thiazolidinone_ hydrogen bonds (Table S11), forming 2D networks. The involvement of the nitrate groups in hydrogen bonding converts the layers into a 3D network.

In addition, powder X-ray diffraction measurements for the main compounds **1**, **2**, **3** and **6·**H_2_O (Figures S5 and S6) were also performed, and these reveal that the isolated products were obtained with high purity, as no additional peaks were observed in the experimental diffractograms.

### Photophysical Studies

The UV–Vis absorption spectra of the three ligands were recorded in methanol over a concentration range of 10^–4^–5 × 10^–6^ M (Figure S7). All spectra exhibit two intense absorption bands with maxima at *ca*. 220 and 320 nm (Figure 6a and Table 1) and high molar extinction coefficients (log ε = 4.21–4.50, Table 1). These features can be attributed to π→π* transitions of the aromatic systems present in the ligands.^39^

**Figure 6:**
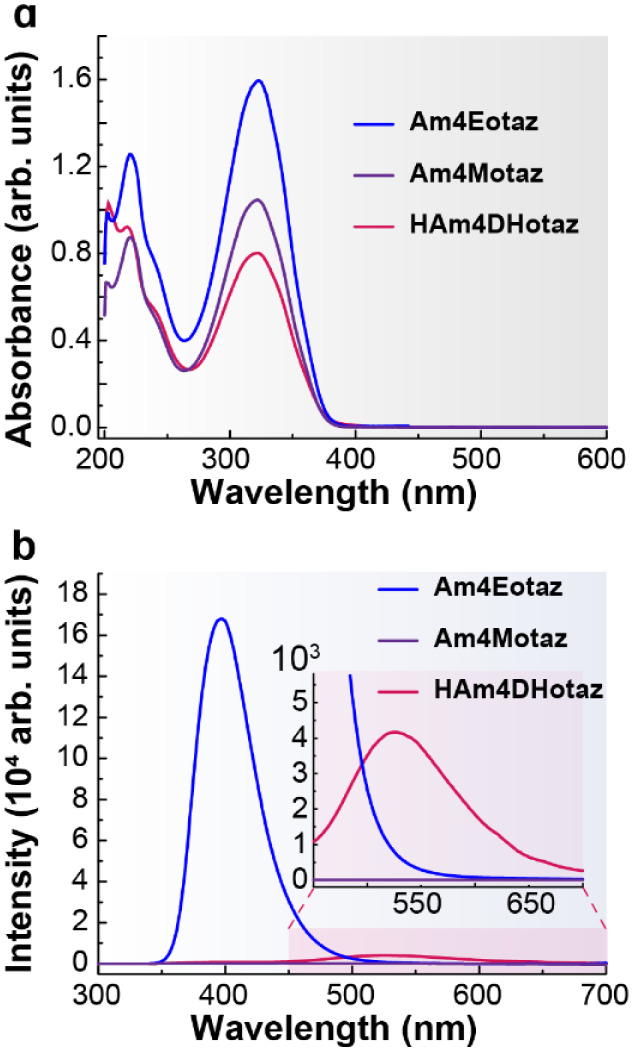
a) Absorption spectra of the ligands in MeOH at 298 K (5 × 10^–5^ M). b) Emission spectra of the ligands in MeOH at 298 K (5 × 10^–5^ M, λ_ex_ = 320 nm). Inset: enlarged view of the 450–700 nm region.

**Table 1:** Photophysical Data of Metal Complexes in Methanol at 298 K: Absorption (λ_abs_) and Emission (λ_em_) Maxima in nm, Molar Absorption Coefficients (log ε), and Stokes Shifts (nm)

	Absorption		Emission	
Compound	λ_abs_/nm	log ε	λ_em_/nm (λ_ex_ = 320 nm)	Stokes shifts/nm
HAm4DHotaz	221	4.27	526	203
	323	4.21		
Am4Motaz	221	4.24	-	-
	323	4.32		
Am4Eotaz	221	4.50	398	75
	323	4.40		
**1**	218	4.56	-	-
	323	4.56		
**2**	219	4.54	-	-
	318	4.54		
**3**	219	4.48	398	74
	324	4.51		
**6**·H_2_O	219	3.99	-	-
	324	3.83		

The band at 323 nm displays a progressive increase in intensity depending on the R group, following the trend R = H < R = CH_3_ < R = CH_2_CH_3_. This behavior is consistent with the electron-donating character of the alkyl substituents, which increases the electron density of the conjugated system and thereby enhances the electronic transition responsible for this absorption band.^40^

The emission spectra of the three ligands were recorded in methanol (Figure 6b, Figure S8 and Table 1) using concentrations from 1 × 10^–4^ M to 5 × 10^–6^ M. For each ligand, emission measurements were performed by exciting at the wavelengths corresponding to their absorption maxima (*ca*. 220 and 320 nm) in order to determine whether the compounds were emissive and to identify the excitation wavelength that produced the strongest emission.

For the ligand HAm4DHotaz (R = H), comparison of the two excitation wavelengths revealed that λ_exc_ = 320 nm yielded the highest emission intensity. This ligand displayed a broad emission band with a maximum at 526 nm and a shoulder at shorter wavelength, around 400 nm (Figure 6b, Figure S8a and Table 1). The emission intensity increased steadily with concentration, with no evidence of reabsorption, reaching its highest value at the most concentrated solution (1 × 10^–4^ M, Figure S8a).

In contrast, the ligand Am4Motaz (R = CH_3_) did not exhibit significant fluorescence under either excitation wavelength. As shown in Figure 6b and Figure S8b, the spectrum displayed no measurable emission. This behavior may arise from the methyl substituent promoting nonradiative deactivation pathways or altering the electronic structure in a way that disfavors fluorescence.

For Am4Eotaz (R = CH_2_CH_3_), the emission spectra (Figure 6b, Figure S8c and Table 1) revealed that excitation at 320 nm resulted in a much stronger signal than excitation at 220 nm; consequently, 320 nm was selected for all measurements. This ligand showed a broad emission band centered around 400 nm. The fluorescence intensity increased progressively with concentration, reaching a maximum at 5 × 10^–5^ M. However, for the most concentrated solution (1 × 10^–4^ M, Figure S8c), the emission intensity decreased, likely due to a reabsorption effect. In any case, this is the ligand that displays the most intense emission (Figure 6b), underscoring the critical influence of the thiazolidinone nitrogen substituent on the fluorescence behavior of this class of organic derivatives. Moreover, the solution behavior of HAm4DHotaz and Am4Eotaz reveals a further significant difference. HAm4DHotaz displays a large Stokes shift of 203, indicative of pronounced excited-state relaxation, likely associated with substantial electronic and/or structural reorganization. In contrast, Am4Eotaz exhibits a much smaller Stokes shift of 75, consistent with a more limited relaxation of the excited state and a closer resemblance between the absorbing and emitting states.

The emission observed in methanol solution for HAm4DHotaz and Am4Eotaz stands in clear contrast to their behavior in the solid state, where no luminescence had previously been reported.^11^

In addition, many studies show that d^10^ metals such as Zn^2+^, Cu^+^ and Ag^+^ engender highly ligand-based luminescent coordination complexes when paired with ligands bearing C=N and/or N-heterocyclic functionalities, as we have also consistently observed in our own work.^41–43^

Thus, the absorption and emission spectra of the four main products **1**, **2**, **3** and **6**·H_2_O were also recorded, in order to see the effect of the coordination of Ag^+^ on the luminescence of the ligands.

The electronic absorption spectra for dilute solutions of all the metal complexes (5 × 10^–5^ M) in methanol show similar features to that of the free ligands (Figure 7a), with similar molar extinction coefficients (Table 1). In this way, two clear absorption bands are observed at *ca*. 220 and 320 nm, attributed to π–π* electronic transitions of the aromatic ring and double bonds of the ligands, as stated.

**Figure 7:**
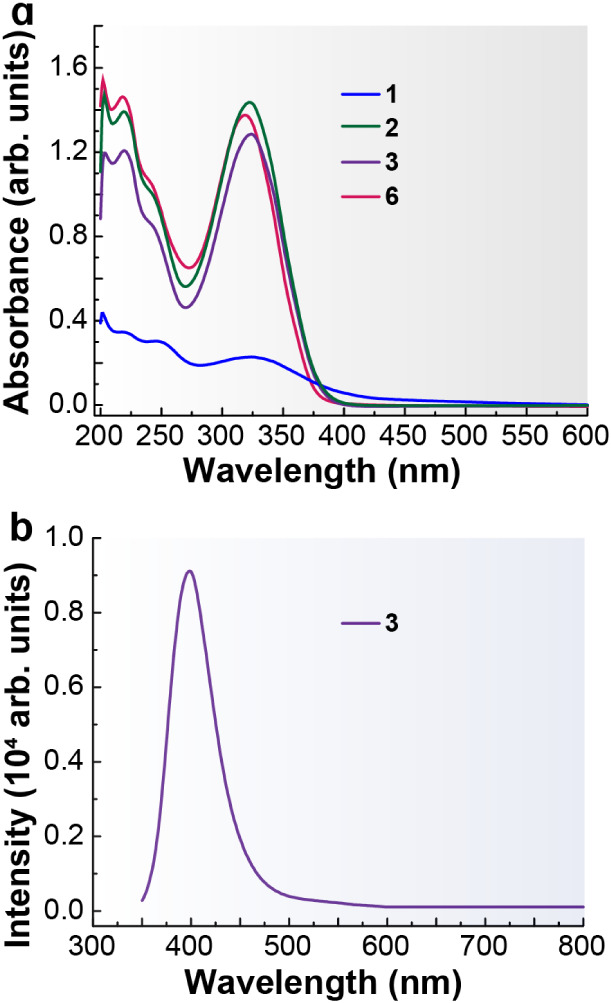
a) Absorption spectra of the complexes in MeOH at 298 K (5 × 10^–5^ M). b) Emission spectra of complex 3 in MeOH at 298 K (5 × 10^–5^ M) recorded at λ_ex_ = 320 nm.

The fluorescence spectra of the compounds (Figure 7b) exhibit clear similarities, although noticeable differences also arise depending on the ligand. Thus, in none of the cases does coordination to Ag^+^ enhance the ligand-centered emission. Accordingly, complex **1** is non-emissive, demonstrating that coordination of Ag^+^ to HAm4DHotaz completely quenches the ligand emission. The perchlorate complex **2** and the nitrate complex **6**·H_2_O are likewise non-emissive at any of their absorption maxima, just as the free ligand Am4Motaz is. These latter results also indicate that the counterion has no discernible influence on the fluorescence behavior of the Am4Motaz-derived complexes.

Complex **3** displays an intense and broad emission band centered at 400 nm (Figure 7b). Its parent ligand, Am4Eotaz, is strongly emissive in solution (Figure 6b), yet upon coordination to Ag^+^ in **3**, the emission is significantly quenched. This behavior suggests that the metal center perturbs the excited-state dynamics of the ligand, suppressing radiative decay pathways that are otherwise active in the free ligand.

Nonetheless, **3** is the only emissive Ag^+^ complex, highlighting the critical influence of the R substituent on the thiazolidinone nitrogen atom on the luminescent properties of these compounds. This observation is particularly significant in the context of thiazolidinone-based metallodrugs, where even a subtle change in the nitrogen substituent can completely quench or enable luminescence.

## Conclusions

The products obtained from the reactions of the thiazolidinone-containing ligands HAm4DHotaz, Am4Motaz, and Am4Eotaz with AgClO_4_ are strongly influenced by the substituent bound to the thiazolidinone N atom. The unsubstituted ligand HAm4DHotaz favors the formation of the polymeric chain {[Ag­(HAm4DHotaz)]­(ClO_4_)}_*n*_ (**1**) whereas the methyl- and ethyl-substituted ligands (Am4Motaz and Am4Eotaz) preferentially form discrete mononuclear [Ag­(L)_2_]­(ClO_4_) complexes (L = Am4Motaz, **2**; L = Am4Eotaz, **3**). Replacement of AgClO_4_ by AgNO_3_ leads to the analogous mononuclear compound [Ag­(Am4Motaz)_2_]­(NO_3_)·H_2_O (**6**·H_2_O), indicating that non-basic counterions plays only a minor role in determining the nature of the isolated products. In addition to the main mononuclear species, several polymeric coordination compounds, {[Ag­(Am4Motaz)­(ClO_4_)]}_*n*_ (**4**), {[Ag_2_(Am4Eotaz)_2_(H_2_O)­(ClO_4_)]­(ClO_4_)·H_2_O}_*n*_ (**5**·H_2_O) and {[Ag_2_(Am4Motaz)_3_]­(NO_3_)_2_·H_2_O}_*n*_ (**7**·H_2_O), can be isolated as byproducts in the obtaining of the mononuclear complexes. The crystal structures of all these complexes show five distinct coordination modes across the series. The mononuclear compounds consistently adopt the common κ^2^NN′ mode, while coordination polymers exhibit four different bridging arrangements, including the unprecedented μ_2_-κ^2^NN′:κ^1^N″ and μ_2_-κ^1^N:κ^2^N′S modes observed in **1** and **4**, and **7**·H_2_O, respectively. Additionally, **5**·H_2_O and **7**·H_2_O display μ_2_-κ^2^NN′:κ^1^S and/or μ_2_-κ^3^NN′S:κ^1^S scarcely related modes. Overall, these results expand the structural diversity known for this family of complexes, highlighting the critical role of N-substitution in steering aggregation toward either mononuclear or polymeric architectures. Furthermore, the identification of novel coordination modes provides a valuable platform for future investigations into the relationship between coordination diversity and biological activity, which appears particularly promising for this class of compounds. In addition, photophysical studies in solution show that the substituent on the thiazolidinone N atom also plays crucial role in the luminescence of the ligands, with the ethyl-substituted Am4Eotaz exhibiting the most intense emission. Coordination of Ag^+^ to the ligands generally quenches luminescence, with only complex **3**, containing Am4Eotaz, remaining emissive. These findings could be particularly relevant for the design of potential bioactive Ag^+^ luminescent metallodrugs, as even subtle modifications of the nitrogen substituents can effectively switch luminescence on or off.

## Supplementary Material



## Notes

Additional figures, X-ray tables, SHAPE calculations, IR, and ^1^H and ^13^C NMR spectra (PDF)
