# Supplementary material for: Coordination Versatility of Thiazolidinone-Based Ligands toward Ag + : Structural and Photophysical Insights
Source: ACS Omega. 2026 Apr 15;11(16):24403–13. doi: 10.1021/acsomega.6c00026 (PMC13130122; doi:10.1021/acsomega.6c00026)
Supplement: Supplementary file 1 [file ao6c00026_si_001.pdf]

Supporting Information

Coordination Versatility of Thiazolidinone-Based Ligands toward Ag^{+}: Structural and Photophysical Insights

Julio Corredoira-Vázquez[£,ε] *Manuel Saa,*[§] *Isabel García-Santos,*[§,*] *Alfonso Castiñeiras,*[§] *and Matilde Fondo*[£,*]

[£] Departamento de Química Inorgánica, Facultade de Química, Universidade de Santiago de Compostela, Campus Vida, 15782 Santiago de Compostela, Spain. E mail: matilde.fondo@usc.es.

[ε] Institute of Materials (iMATUS), Universidade de Santiago de Compostela, 15782 Santiago de Compostela, Spain.

[§] Departamento de Química Inorgánica, Facultade de Farmacia, Universidade de Santiago de Compostela, Campus Vida, 15782 Santiago de Compostela, Spain. E mail: isabel.garcia@usc.es.

Table S1. Crystal data and structure refinement for silver complexes **1-7**·H_2O. **3**

Figure S1. IR spectra for {[Ag(HAm4DHotaz)](ClO_4)}$_n$ (a), Ag(Am4Motaz)$_2$](ClO_4) (b), [Ag(Am4Eotaz)$_2$](ClO_4)] (c) and [Ag(Am4Motaz)$_2$](NO_3)·H_2O (d). **5**

Figure S2. ^{1}H NMR spectra for {[Ag(HAm4DHotaz)](ClO_4)}$_n$ (a), Ag(Am4Motaz)$_2$](ClO_4) (b), [Ag(Am4Eotaz)$_2$](ClO_4)] (c) and [Ag(Am4Motaz)$_2$](NO_3)·H_2O (d). **7**

Figure S3. ^{13}C NMR spectra for {[Ag(HAm4DHotaz)](ClO_4)}$_n$ (a), Ag(Am4Motaz)$_2$](ClO_4) (b), [Ag(Am4Eotaz)$_2$](ClO_4)] (c) and [Ag(Am4Motaz)$_2$](NO_3)·H_2O (d). **9**

Figure S4. Molecular structure for: a) [Ag(Am4Motaz)$_2$](ClO_4) (**2**); b) [Ag(Am4Motaz)$_2$](NO_3) in [Ag(Am4Motaz)$_2$](NO_3)·H_2O (**6**·H_2O). **11**

Table S2. Main bond distances (Å) and angles (°) for [Ag(Am4Motaz)$_2$](ClO_4) (**2**), [Ag(Am4Eotaz)$_2$](ClO_4) (**3**) and [Ag(Am4Motaz)$_2$](NO_3)·H_2O (**6**·H_2O). **12**

Table S3. Classical hydrogen bonds [Å,º] for [Ag(Am4Motaz)$_2$](ClO_4) (**2**), [Ag(Am4Eotaz)$_2$](ClO_4) (**3**) and [Ag(Am4Motaz)$_2$](NO_3)·H_2O (**6**·H_2O) **12**

Table S4. Main bond distances (Å) and angles (°) for {[Ag(HAm4DHotaz)](ClO_4)}$_n$ (**1**) and {[Ag(Am4Motaz)(ClO_4)]}$_n$ (**4**). **13**

Table S5. SHAPE v2.1. Continuous Shape Measures Calculation (c) 2013, Electronic Structure Group, Universitat de Barcelona, for {[Ag(HAm4DHotaz)](ClO_4)}$_n$ (**1**) and {[Ag(Am4Motaz)(ClO_4)]}$_n$ (**4**). **13**

Table S6. Classical hydrogen bonds [Å,º] for [Ag(HAm4DHotaz)](ClO_4)}$_n$ (**1**) and {[Ag(Am4Motaz)(ClO_4)]}$_n$ (**4**). **14**

Table S7. Main bond distances (Å) and angles (°) for {[Ag_2($Am4Eotaz)_2$(H_2O)(ClO_4)](ClO_4)·H_2O}$_n$ (**5**·H_2O). **14**

Table S8. Classical hydrogen bonds [Å,º] for {[Ag_2($Am4Eotaz)_2$(H_2O)(ClO_4)](ClO_4)·H_2O}$_n$ (**5**·H_2O) **14**

Table S9. Main bond distances (Å) and angles (°) for {[Ag_2($Am4Motaz)_3$]($NO_3)_2$·H_2O}$_n$ (**7**·H_2O) **15**

Table S10. SHAPE v2.1. Continuous Shape Measures Calculation (c) 2013, Electronic Structure Group, Universitat de Barcelona, for {[Ag_2($Am4Motaz)_3$]($NO_3)_2$·H_2O}$_n$ (**7**·H_2O). **15**

Table S11. Classical hydrogen bonds [Å,º] for {[Ag_2Am4$Motaz_3$]($NO_3)_2$·H_2O}$_n$ (7·H_2O) **16**

Figure S5. Comparative powder X-ray diffractograms for: up) **1** (blue) and the simulation from single X-ray diffraction data (red). Bottom) **2** (blue) and the simulation from single X-ray diffraction data (red). **17**

Figure S6. Comparative powder X-ray diffractograms for: up) **3** (blue) and the simulation from single X-ray diffraction data (red). Bottom) **6**·H_2O (blue) and the simulation from single X-ray diffraction data (red). **18**

Figure S7. UV–Vis spectra of the ligands (a) HAm4DHotaz, (b) Am4Motaz, and (c) Am4Eotaz in MeOH at different concentrations, recorded at room temperature. **19**

Figure S8. Emission spectra in MeOH recorded at room temperature for (a) HAm4DHotaz at different concentrations with λ_{ex} = 320 nm; (b) Am4Motaz at a concentration of $1\cdot10^{-4}$ M and at different excitation wavelengths (λ_{ex} = 220 nm and λ_{ex} = 320 nm); and (c) Am4Eotaz at different concentrations with λ_{ex} = 320 nm. **20**

Table S1. Crystal data and structure refinement for silver complexes **1**-**7**·H_2O

	{[Ag(Am4DHotaz)](ClO_4)}$_n$ (**1**)	[Ag($Am4Motaz)_2$](ClO_4) (**2**)	[Ag($Am4Eotaz)_2$](ClO_4) (**3**)	{[Ag(Am4Motaz)(ClO_4)]}$_n$ (**4**)
Empirical formula	$C_9H_9AgClN_5O_5S$	$C_{20}H_{22}AgClN_{10}O_6S_2$	$C_{22}H_{26}AgClN_{10}O_6S_2$	$C_{10}H_{11}AgClN_5O_5S$
Formula weight	442.59	705.91	733.97	456.62
Temperature/K	100(2)	100(2)	100(2)	100(2)
Wavelength/Å	0.71073	0.71073	0.71073	0.71073
Crystal system	Monoclinic	Triclinic	Triclinic	Monoclinic
Space group	*P2₁/c*	*P_1*	*P_1*	*P2₁/c*
a/Å	9.4347(7)	9.0993(2)	11.0267(4)	12.0086(3)
b/Å	15.3788(13)	12.9843(3)	11.1989(4)	8.2658(2)
c/Å	9.5639(8)	13.1418(3)	13.5331(5)	15.4589(4)
α/º	90	108.2250(10)	106.208(2)	90
β/º	101.678(3)	93.7630(10)	101.949(2)	109.4070(10)
γ/º	90	93.1820(10)	111.447(2)	90
Volume/$Å^{-3}$	1358.94(19)	1466.87(6)	1401.45(9)	1447.28(6)
Z	4	2	2	4
Calc. density/g/m^3	2.163	1.743	1.739	2.096
Absorp. coefc./mm^{-1}	1.866	0.986	1.022	1.756
Crystal size/mm	0.380 x 0.020 x 0.010	0.400 x 0.220 x 0.170	0.180 x 0.060 x 0.050	0.120 x 0.080 x 0.030
ϑ range/º	2.55 / 26.35	1.64 / 26.59	1.67 / 26.02	1.80 / 26.37
Refl. collect/unique	2713 / 2713	6068 / 6068	5438 / 5438	2950 / 2950
Data/parameters	2713 / 193	6068 / 397	5438 / 379	2950 / 208
Goodness-of-fit on F^2	0.962	1.046	1.023	1.073
Final *R* indices	R1 = 0.0481 wR2 = 0.0929	R1 = 0.0275 wR2 = 0.0651	R1 = 0.0357 wR2 = 0.0601	R1 = 0.0264 wR2 = 0.0620
R indices (all data)	R1 = 0.0925 wR2 = 0.1038	R1 = 0.0307 wR2 = 0.0666	R1 = 0.0627 wR2 = 0.0681	R1 = 0.0371 wR2 = 0.0652

Continuation Table S1. Crystal data and structure refinement for silver complexes **1**-**7**·H_2O

	{[Ag_2(Am4Eotaz)$_2$(H_2O)(ClO_4)](ClO_4)·H_2O}$_n$ (**5**·H_2O)	[Ag(Am4Motaz)$_2$](NO_3)·H_2O (**6**·H_2O)	{[Ag_2(Am4Motaz)$_3$](NO_3)$_2$·H_2O}$_n$ (**7**·H_2O)
Empirical formula	$C_{22}H_{30}Ag_2Cl_2N_{10}O_{12}S_2$	$C_{20}H_{24}AgN_{11}O_6S_2$	$C_{30}H_{29}Ag_2N_{17}O_{10}S_3$
Formula weight	977.32	686.49	1099.62
Temperature/K	100(2)	100(2)	100(2)
Wavelength/Å	0.71073	0.71073	0.71073
Crystal system	Orthorhombic	Triclinic	Monoclinic
Space group	*Pna21*	*P_1*	*C2/c*
a/Å	13.5713(3)	6.944(2)	48.5513(15)
b/Å	14.0338(3)	12.227(4)	7.4503(2)
c/Å	17.9103(3)	16.271(5)	24.6175(8)
α/º	90	100.501(5)	90
β/º	90	102.140(5)	110.4370(10)
γ/º	90	101.782(5)	90
Volume/$Å^{-3}$	3411.14(12)	1285.0(7)	8344.2(4)
Z	4	2	8
Calc. density/g/m^3	1.903	1.824	1.751
Absorp. coefc./mm^{-1}	1.501	1.111	1.164
Crystal size/mm	0.230 x 0.170 x 0.070	0.330 x 0.300 x 0.060	0.22 x 0.09 x 0.08
ϑ range/º	1.84 / 28.29	1.75 / 25.42	0.90 / 28.40
Refl. collect/unique	7986 / 7986	4690 / 4690	10336 / 10336
Data/parameters	7988 / 452	4690 / 361	10336 / 559
Goodness-of-fit on F^2	1.050	0.948	1.074
Final *R* indices	R1 = 0.0413 wR2 = 0.1020	R1 = 0.0357 wR2 = 0.0878	R1 = 0.0573 wR2 = 0.1413
R indices (all data)	R1 = 0.0502 wR2 = 0.1063	R1 = 0.0466 wR2 = 0.0927	R1 = 0.0661 wR2 = 0.1492

a

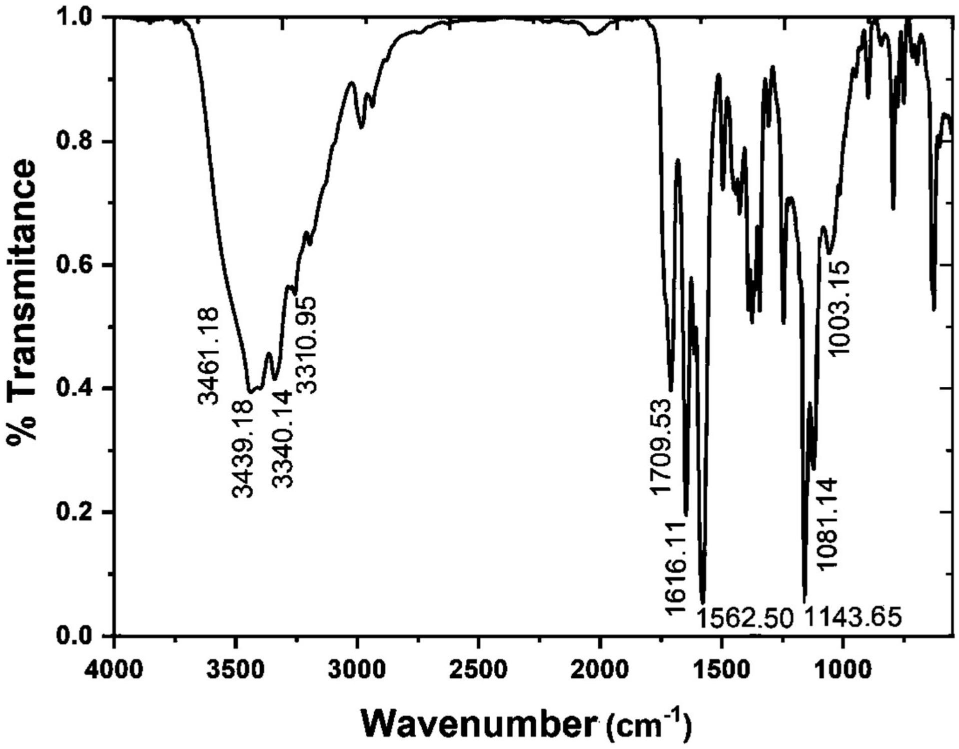

% Transmitance
Wavenumber (cm-1)
3461.18
3439.18
3340.14
3310.95
1709.53
1616.11
1562.50
1143.65
1081.14
1003.15

b

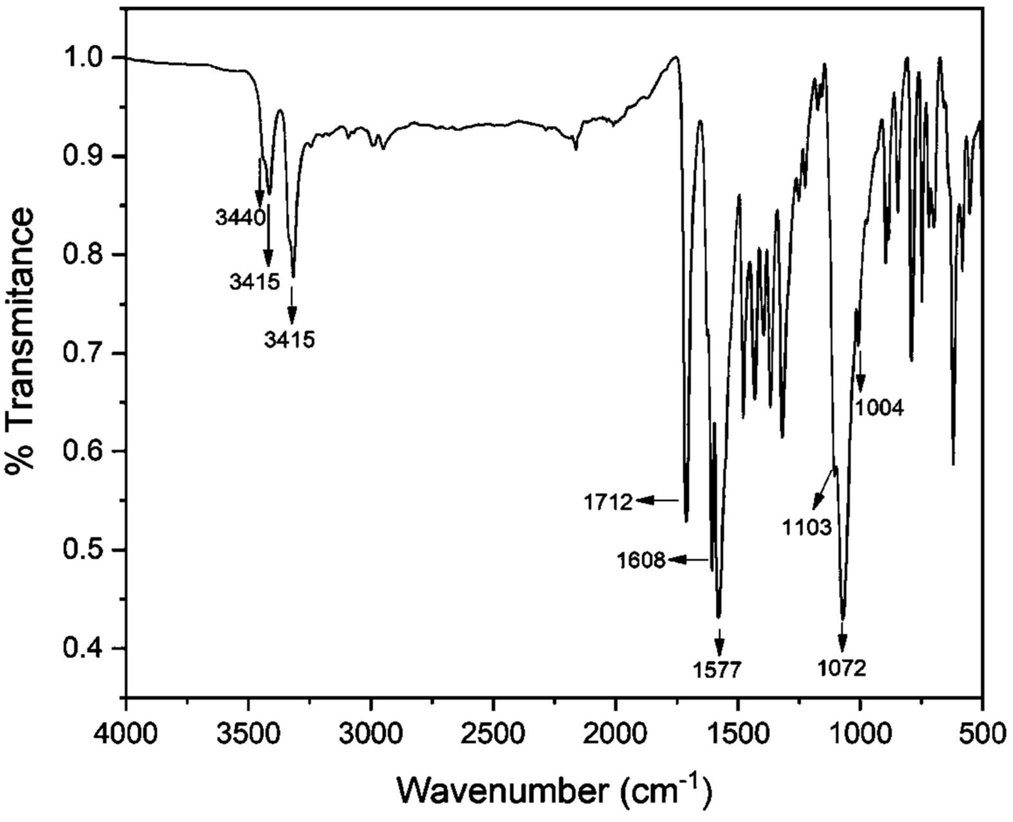

% Transmitance
Wavenumber (cm-1)
3440
3415
3415
1712
1608
1577
1103
1072
1004

c

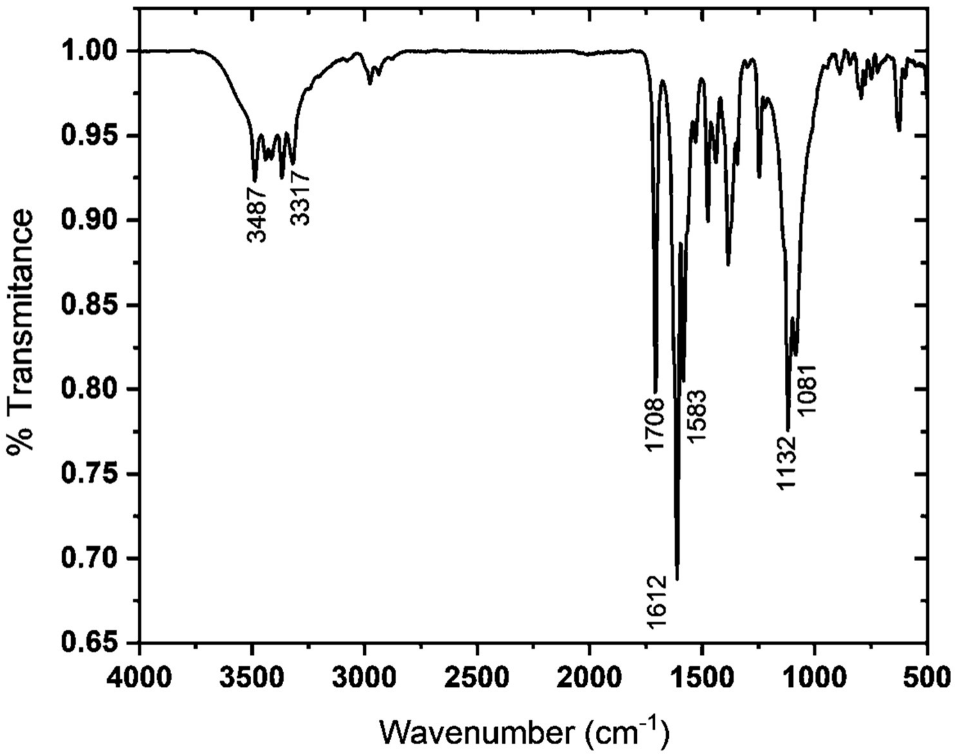

d

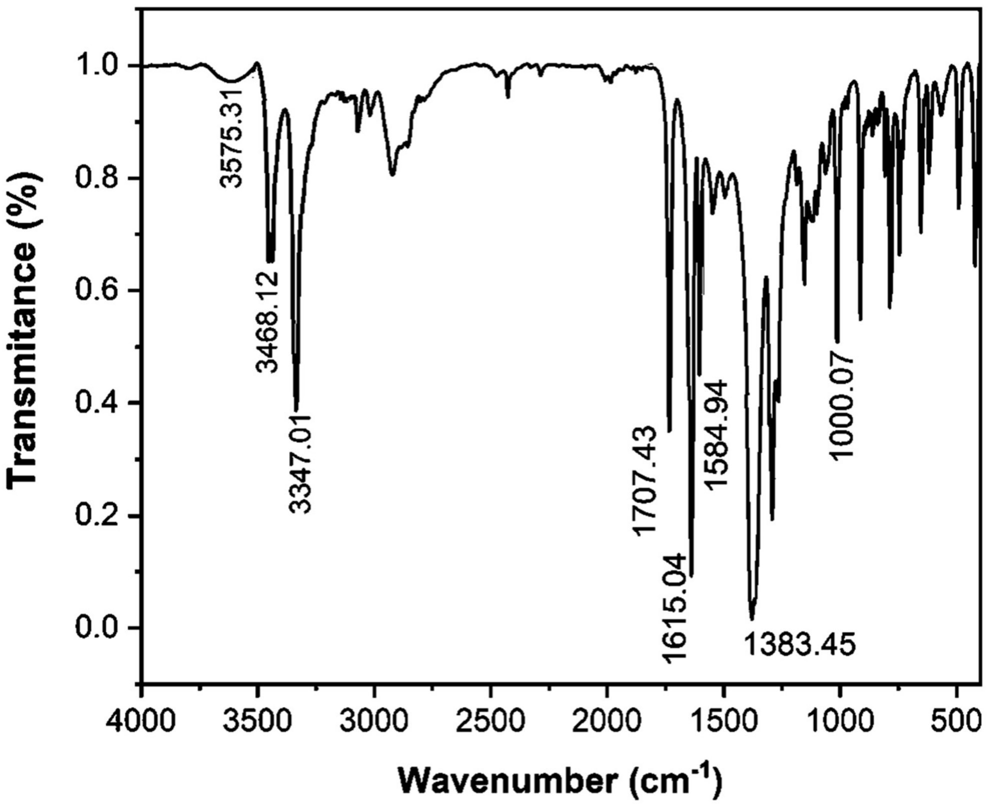

Figure S1. IR spectra for {[Ag(HAm4DHotaz)](ClO$_4$)}$_n$ (a), Ag(Am4Motaz)$_2$](ClO$_4$) (b), [Ag(Am4Eotaz)$_2$](ClO$_4$)] (c) and [Ag(Am4Motaz)$_2$](NO$_3$)·H$_2$O (d).

a

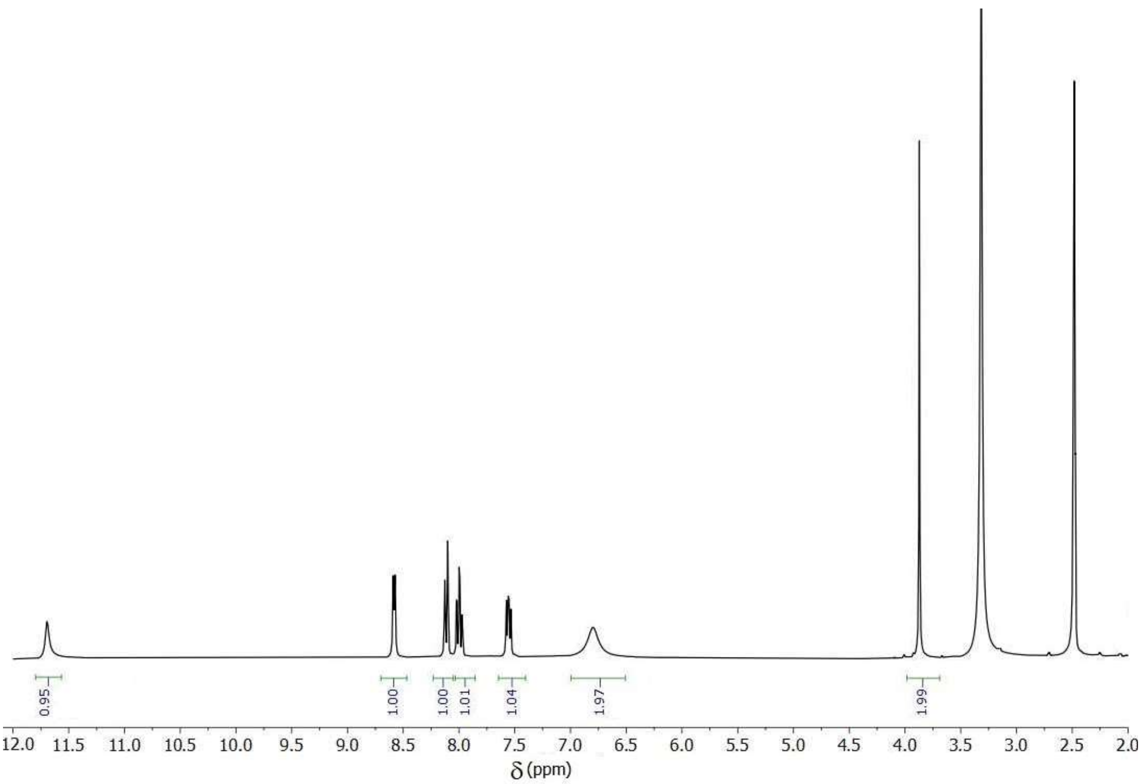

0.95
1.00
1.00
1.01
1.04
1.97
1.99
12.0 11.5 11.0 10.5 10.0 9.5 9.0 8.5 8.0 7.5 7.0 6.5 6.0 5.5 5.0 4.5 4.0 3.5 3.0 2.5 2.0
δ (ppm)

b

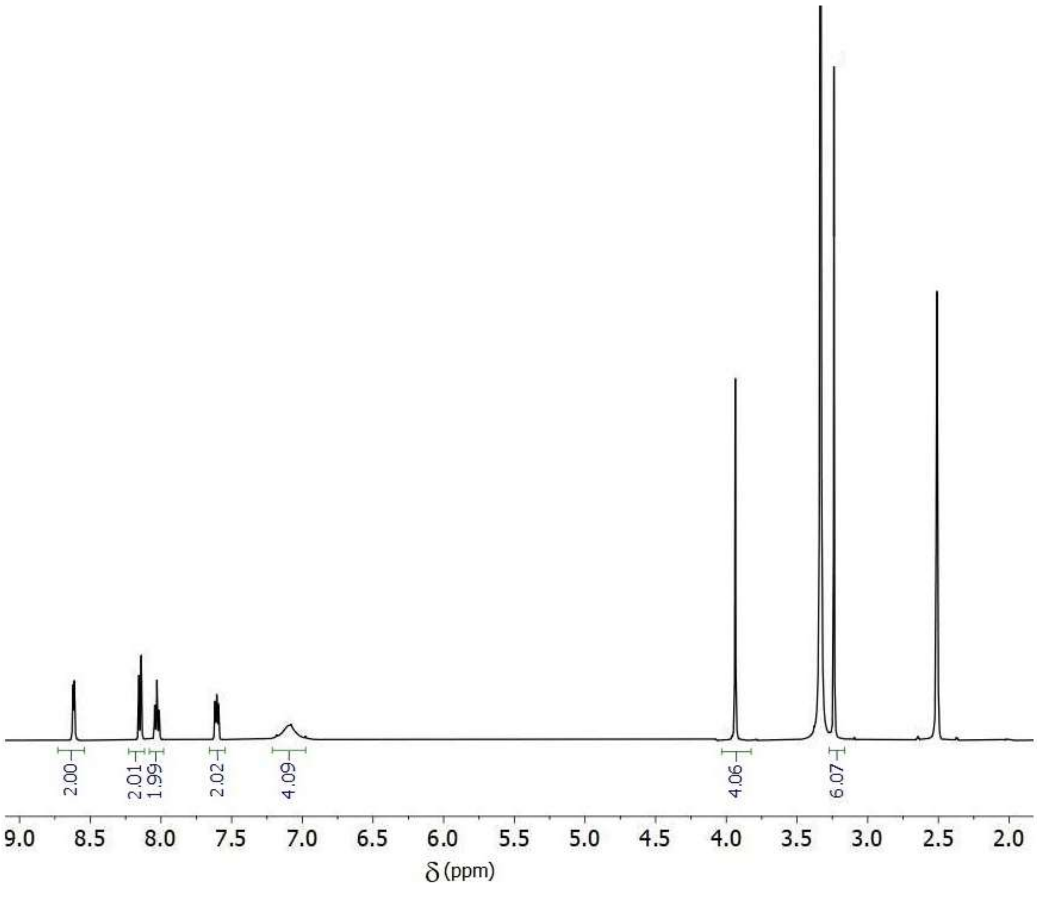

2.00
2.01
1.99
2.02
4.09
4.06
6.07
9.0 8.5 8.0 7.5 7.0 6.5 6.0 5.5 5.0 4.5 4.0 3.5 3.0 2.5 2.0
δ (ppm)

c

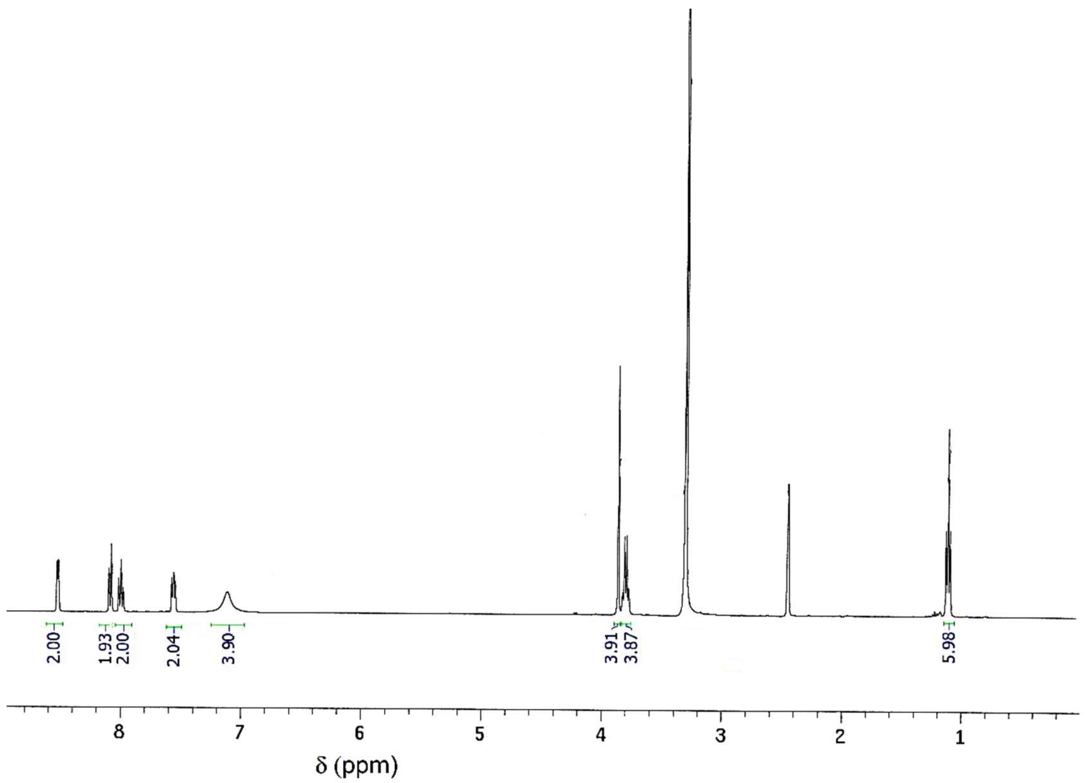

d

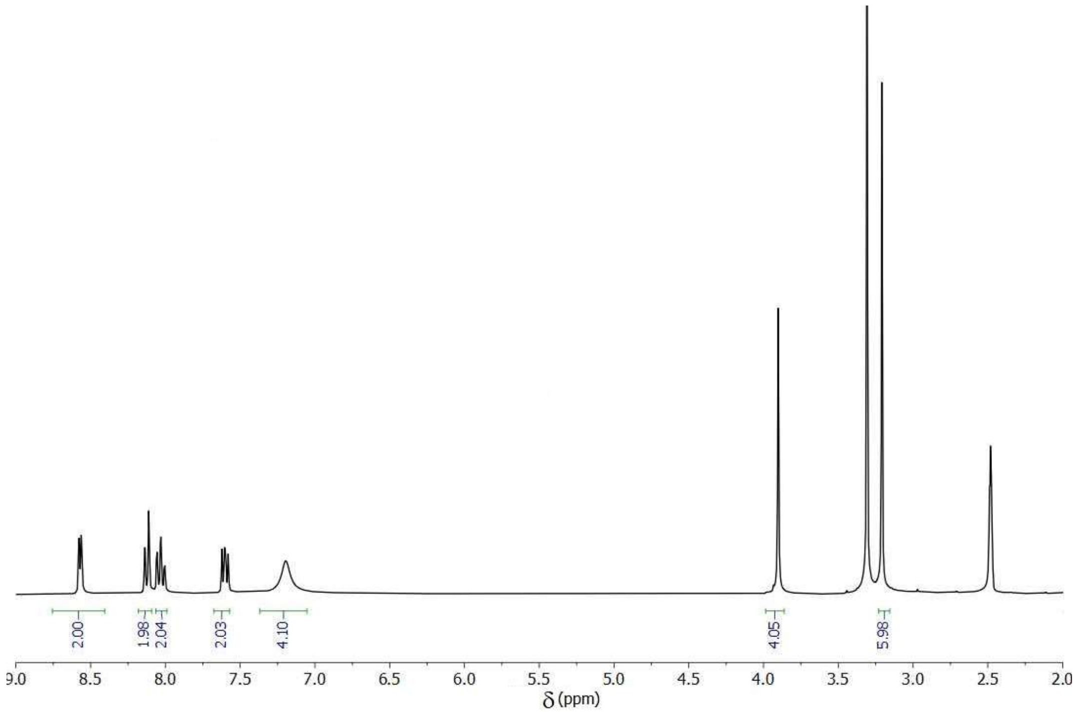

Figure S2. ^{1}H NMR spectra for {[Ag(HAm4DHotaz)](ClO$_4$)}$_n$ (a), Ag(Am4Motaz)$_2$](ClO$_4$) (b), [Ag(Am4Eotaz)$_2$](ClO$_4$)] (c) and [Ag(Am4Motaz)$_2$](NO$_3$)·H$_2$O (d).

a

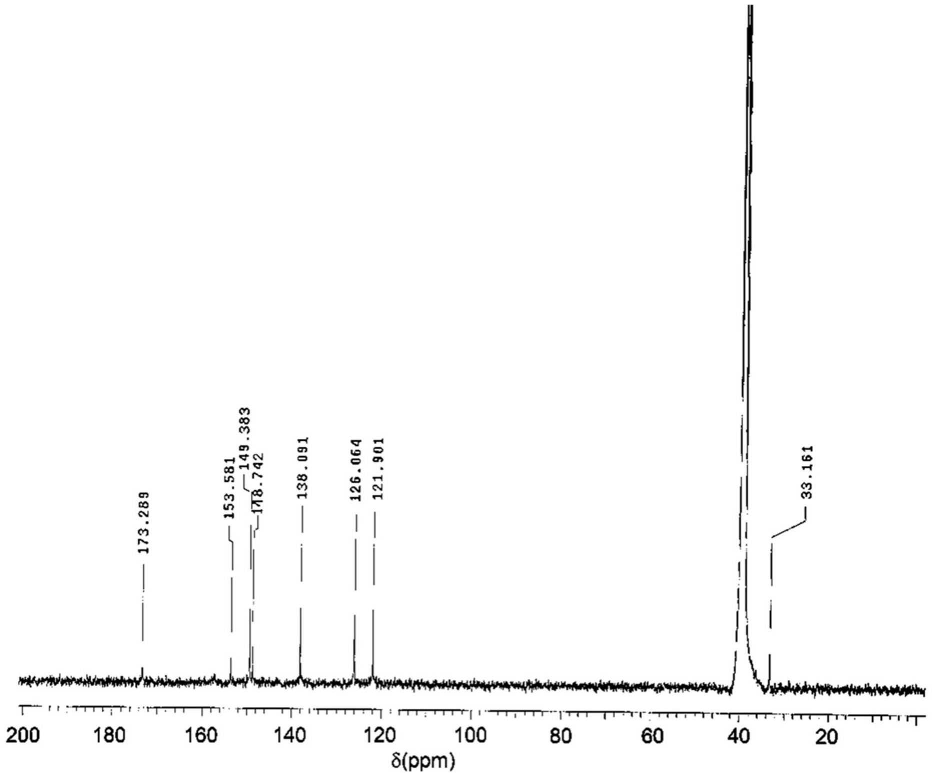

173.289
153.581
149.383
148.742
138.091
126.064
121.901
33.161
200
180
160
140
120
100
80
60
40
20
δ(ppm)

b

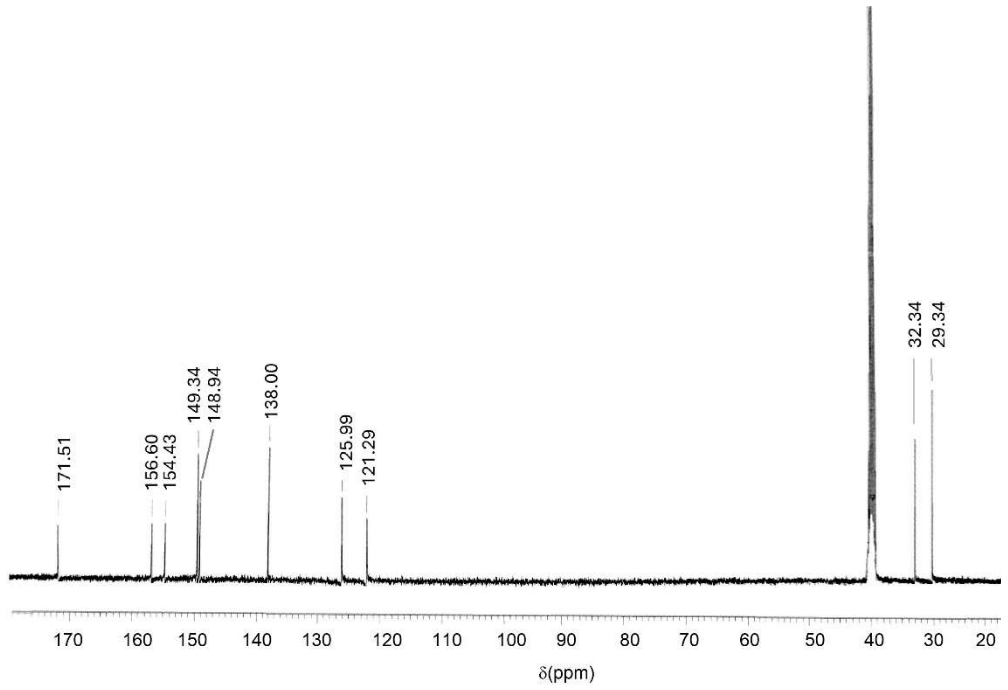

171.51
156.60
154.43
149.34
148.94
138.00
125.99
121.29
32.34
29.34
170
160
150
140
130
120
110
100
90
80
70
60
50
40
30
20
δ(ppm)

c

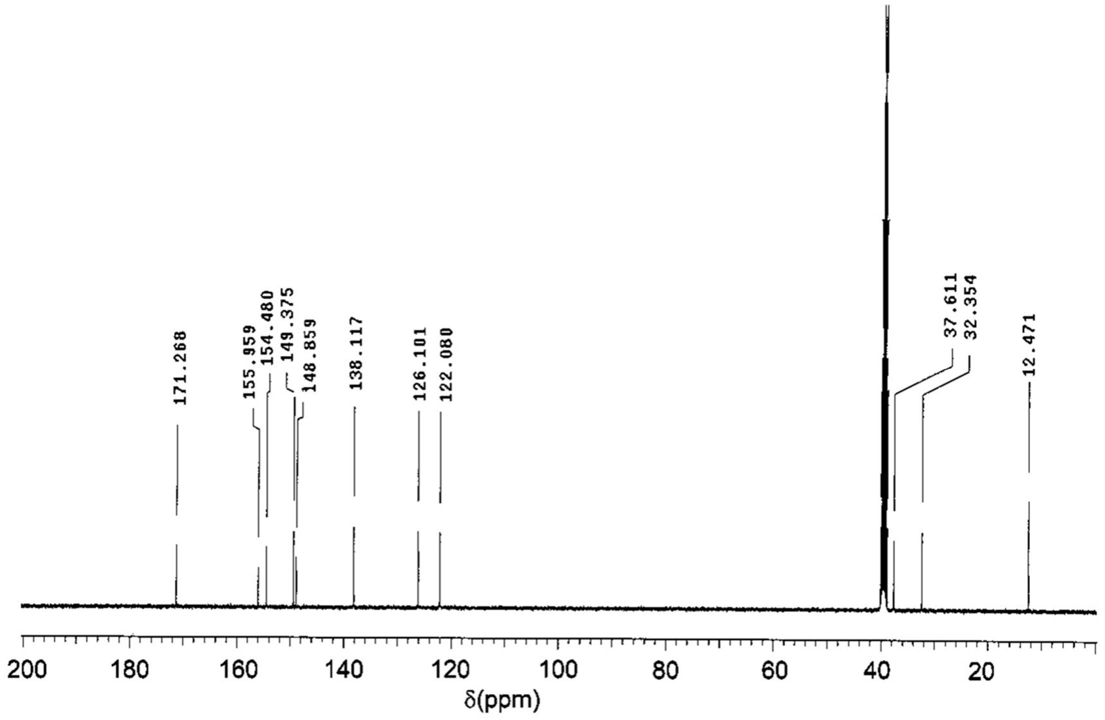

d

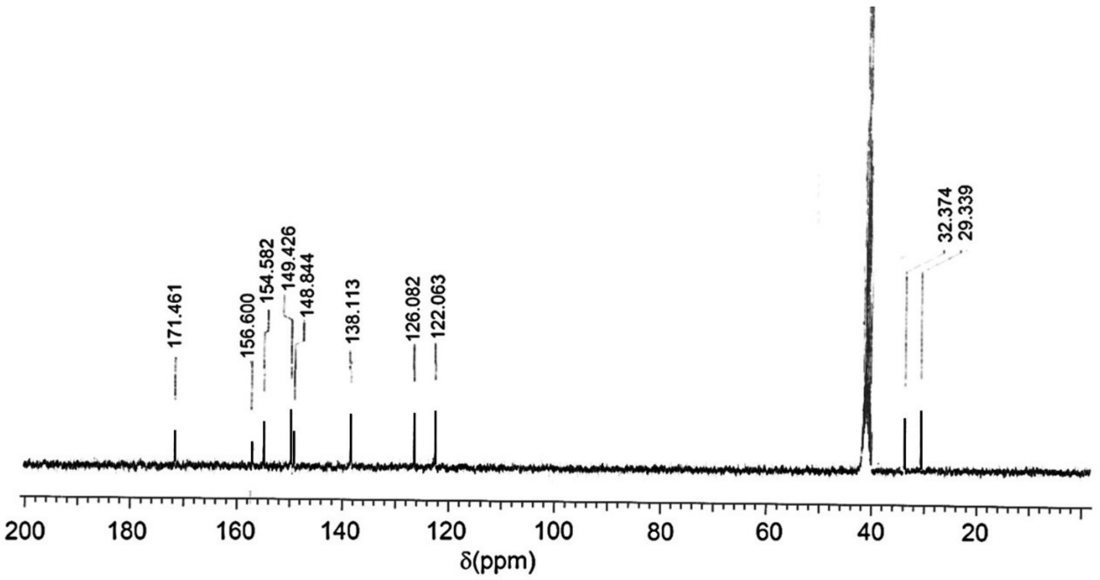

Figure S3. ^{13}C NMR spectra for $\{[Ag(HAm4DHotaz)](ClO_4)\}_n$ (a), $Ag(Am4Motaz)_2](ClO_4)$ (b), $[Ag(Am4Eotaz)_2](ClO_4)]$ (c) and $[Ag(Am4Motaz)_2](NO_3)\cdot H_2O$ (d).

a

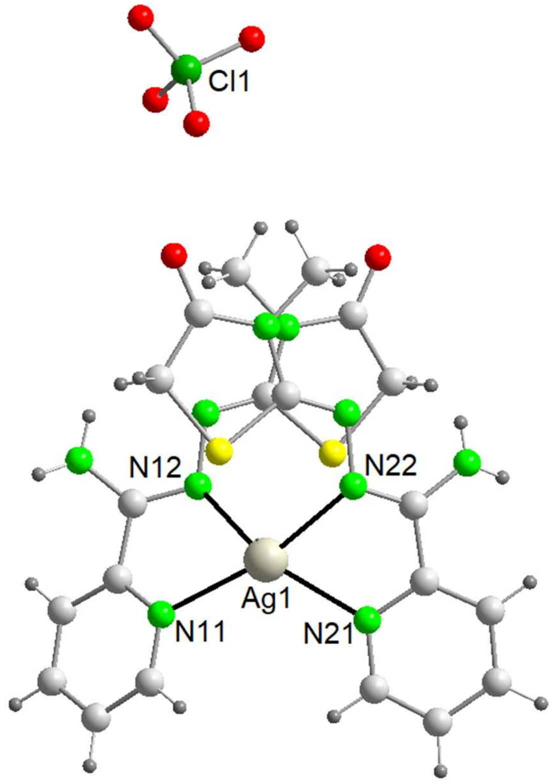

b

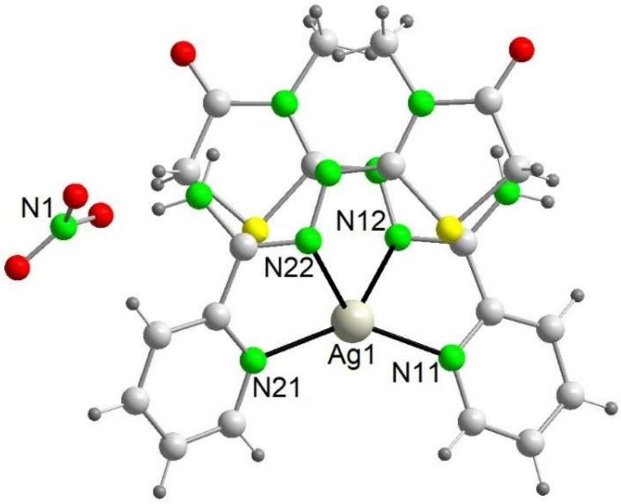

Figure S4. Molecular structure for: a) [Ag(Am4Motaz)$_2$](ClO$_4$) (**2**); b) [Ag(Am4Motaz)$_2$](NO$_3$) in [Ag(Am4Motaz)$_2$](NO$_3$)·H$_2$O (**6**·H$_2$O).

Table S2. Main bond distances (Å) and angles (°) for [Ag(Am4Motaz)$_2$](ClO$_4$) (**2**), [Ag(Am4Eotaz)$_2$](ClO$_4$) (**3**) and [Ag(Am4Motaz)$_2$](NO$_3$)·H$_2$O (**6**·H$_2$O).

	**2**	**3**	**6**·H$_2$O
Ag1-N11	2.299(3)	2.231(3)	2.226(2)
Ag1-N12	2.366(3)	2.570(3)	2.463(2)
Ag1-N21	2.254(3)	2.250(3)	2.254(2)
Ag1-N22	2.468(3)	2.458(3)	2.404(2)
N11-Ag1-N21	133.50(10)	140.53(10)	140.60(8)
N11-Ag1-N22	144.62(9)	142.19(9)	138.43(7)
N21-Ag1-N22	72.23(9)	70.03(9)	72.53(7)
N11-Ag1-N12	71.98(10)	69.99(9)	72.18(7)
N21-Ag1-N12	146.39(11)	144.79(9)	140.11(7)
N22-Ag1-N12	98.17(9)	93.52(8)	95.15(7)

Table S3. Classical hydrogen bonds [Å,º] for [Ag(Am4Motaz)$_2$](ClO$_4$) (**2**), [Ag(Am4Eotaz)$_2$](ClO$_4$) (**3**) and [Ag(Am4Motaz)$_2$](NO$_3$)·H$_2$O (**6**·H$_2$O).

D-H···A	d(D-H)	d(H···A)	d(D···A)	<(DHA)
**[Ag(Am4Motaz)$_2$](ClO$_4$)**				
N15-H15A...O2^1	0.88	2.31	3.133(4)	155.4
N15-H15B...O14	0.88	2.44	3.057(4)	127.4
N25-H25A...O1^2	0.88	2.20	2.935(4)	141.3
N25-H25B...O13^3	0.88	2.26	3.023(4)	145.0
^{1} -x+1,-y+1,-z+1; ^{2} -x+1,-y+2,-z+1; ^{3} x,y+1,z				
**[Ag(Am4Eotaz)$_2$](ClO$_4$)**				
N15-H15A...O12^1	0.88	2.18	3.009(3)	157.1
N15-H15B...O2^2	0.88	2.18	3.020(3)	159.0
N25-H25A...O1^3	0.88	2.29	3.078(3)	149.5
N25-H25B...O11^4	0.88	2.23	3.001(3)	146.2
^{1} -x+2,-y+2,-z+1; ^{2} x,y+1,z ; ^{3} -x+1,-y+1,-z+1; ^{4} x-1,y,z				
**[Ag(Am4Motaz)$_2$](NO$_3$)·H$_2$O**				
N15-H15A...O3^1	0.88	2.00	2.874(3)	174.7
N15-H15B...O2^2	0.82	2.20	3.003(3)	163.5
N25-H25A...O11^3	0.89	2.23	3.092(3)	163.1
N25-H25A...O13^3	0.89	2.42	3.160(3)	140.4
N25-H25B...O1^4	0.87	2.23	2.976(3)	143.3
O3-H3A...O13	0.96	1.91	2.841(3)	162.2
O3-H3B...O11^3	0.83	2.03	2.855(3)	169.6
^{1} x+1,y,z; ^{2} -x+1,-y+1,-z+1; ^{3} -x+1,-y,-z+1; ^{4} x+1,y-1,z				

Table S4. Main bond distances (Å) and angles (°) for {[Ag(HAm4DHotaz)](ClO_4)}$_n$ (**1**) and {[Ag(Am4Motaz)(ClO_4)]}$_n$ (**4**).

	**1**	**4**
Ag1-N13	2.216(5)	2.249(2)
Ag1-N11^1	2.297(5)	2.317(2)
Ag1-N12^1	2.301(5)	2.350(2)
Ag1-O14^1/11^2	3.185(5)	2.740(2)
N13-Ag1-N11^1	126.34(18)	146.14(9)
N13-Ag1-N12^1	162.87(18)	140.92(8)
N11^1-Ag1-N12^1	70.76(17)	70.72(8)
N13-Ag1-O11^2		85.66(8)

^{1} -x,y-1/2,-z+1/2 for {[Ag(HAm4DHotaz)](ClO_4)}$_n$; -x,y-1/2,-z+1/2 for {[Ag(Am4Motaz)(ClO_4)]}$_n$;
^{2}-x,-y+1,-z.

Table S5. SHAPE v2.1. Continuous Shape Measures Calculation (c) 2013, Electronic Structure Group, Universitat de Barcelona, for {[Ag(HAm4DHotaz)](ClO_4)}$_n$ (**1**) and {[Ag(Am4Motaz)(ClO_4)]}$_n$ (**4**).

	**Coordination number 3**	
mvOC-3	4 C2v	mer-Trivacant octahedron
fvOC-3	3 C3v	fac-Trivacant octahedron
vT-3	2 C3v	Vacant tetrahedron
TP-3	1 D3h	Trigonal
	**Coordination number 4**	
vTBPY-4	4 C3v	Axially vacant trigonal bipyramid
SS-4	3 C2v	Seesaw or sawhorse‡ (cis-divacant octahedron)
T-4	2 Td	Tetrahedron
SP-4	1 D4h	Square planar

1

Structure [ML3]	**mvOC-3**	fvOC-3	vT-3	TP-3
,	**4.514**,	18.694,	11.768,	9.264

4

Structure [ML4]	vTBPY-4	**SS-4**	T-4	SP-4
	9.495,	**7.301**,	8.585,	21.460

Table S6. Classical hydrogen bonds [Å,º] for [Ag(HAm4DHotaz)](ClO_4)}$_n$ (**1**) and {[Ag(Am4Motaz)(ClO_4)]}$_n$ (**4**).

D-H···A*	d(D-H)	d(H···A)	d(D···A)	<(DHA)
		**{[Ag(HAmDHotaz)](ClO_4)}$_n$**		
N14-H14A...O13[1]	0.88	2.23	3.043(6)	152.7
N16-H16A...O14[1]	0.88	2.14	2.991(7)	162.6
N16-H16B...O12[3]	0.88	2.30	3.160(7)	166.5
[1] x,-y+1/2,z-1/2; [2] x,-y+1/2,z+1/2; [3] -x+2,y+1/2,-z+1/2				
		**{[Ag(Am4Motaz)(ClO_4)]}$_n$**		
N15-H15A...O11[4]	0.85	2.18	3.025(3)	172.2
N15-H15B...O14[3]	0.88	2.09	2.927(3)	159.8
[3] -x,y+1/2,-z+1/2; [4] x,-y+1/2,z+1/2				

*N15 or N16: NH_2 group; N14: thiazolidinone ring

Table S7. Main bond distances (Å) and angles (°) for {[Ag_2(Am4Eotaz)$_2$(H_2O)(ClO_4)](ClO_4)·H_2O}$_n$ (**5**·H_2O).

Ag1-N12	2.296(6)	Ag2-N22	2.421(6)
Ag1-N11	2.341(6)	Ag2-N21	2.283(6)
Ag1-O21[2]	2.869(14)	Ag2-O3	2.340(6)
Ag1-O22[2]	2.892(9)	Ag2-O22[2]	2.951(8)
Ag1-S1	2.9239(19)	Ag2-S1	2.7181(19)
Ag1-S2[1]	2.5017(18)	Ag1···Ag2	4.2587(7)
Ag1···Ag2[1]	3.3482(7)		
N12-Ag1-N11	70.9(2)	N21-Ag2-N22	70.8(2)
N12-Ag1-S2[1]	142.54(15)	N21-Ag2-O3	132.6(2)
N11-Ag1-S2[1]	128.24(16)	O3-Ag2-N22	137.1(2)
N12-Ag1-S1	64.92(15)	O3-Ag2-S1	91.04(19)
S2[1]-Ag1-S1	108.73(6)	N22-Ag2-S1	100.39(15)
		O22[2]-Ag2-N22	144.8(2)

[1] x-1/2,-y+3/2,z; [2] x+1/2,-y+3/2,z

Table S8. Classical hydrogen bonds [Å,º] for {[Ag_2(Am4Eotaz)$_2$(H_2O)(ClO_4)](ClO_4)·H_2O}$_n$ (**5**·H_2O).

D-H···A*	dD-H	dH···A	dD···A	<DHA
O3-H3B...O4	0.90	1.83	2.67716	155.2
N15-H15B...O2[1]	0.86	2.12	2.9636	167.0
N25-H25A...O12	0.86	2.49	3.1078	129.2
N25-H25B...O1[1]	0.86	2.12	2.9126	152.1
[1] -x+1,-y+1, z-1/2				

*N15 or N26: NH_2 group

Table S9. Main bond distances (Å) and angles (°) for {[Ag_2(Am4Motaz)$_3$](NO_3)$_2$·H_2O}$_n$ (**7**·H_2O).

Ag1-N21	2.257(4)	Ag2-N22	2.251(4)
Ag1-N11	2.304(4)	Ag2-N31^2	2.305(4)
Ag1-N12	2.368(4)	Ag2-N32^2	2.354(4)
Ag1-S3	2.8890(13)	Ag2-S2	2.7749(12)
Ag1···Ag2	2.9401(5)	Ag2-S3^2	3.0072(12)
Ag1···Ag2^1	4.5611(5)		
N21-Ag1-N12	144.66(14)	N22-Ag2-N31^2	150.41(14)
N21-Ag1-N11	140.09(15)	N32^2-Ag2-S2	145.02(11)
N21-Ag1-S3	85.25(10)	N22-Ag2-N32^2	136.77(14)
N11-Ag1-S3	101.08(11)	N31^2-Ag2-N32^2	70.94(15)
N12-Ag1-S3	105.29(10)	N22-Ag2-S2	72.05(10)
N11-Ag1-N12	71.99(14)	N31^2-Ag2-S2	87.65(11)
Ag2-Ag1-Ag2^1	166.313(15)	N22-Ag2-S3^2	82.24(10)
		N31^2-Ag2-S3^2	126.53(10)
		N32^2-Ag2-S3^2	63.77(11)
		S2-Ag2-S3^2	114.27(4)

^{1} x,y+1,z; ^{2} x,y-1,z

Table S10. SHAPE v2.1. Continuous Shape Measures Calculation (c) 2013, Electronic Structure Group, Universitat de Barcelona, for {[Ag_2(Am4Motaz)$_3$](NO_3)$_2$·H_2O}$_n$ (**7**·H_2O).

	**Coordination number 4**	
vTBPY-4	4 C3v	Axially vacant trigonal bipyramid
SS-4	3 C2v	Seesaw or sawhorse‡ (cis-divacant octahedron)
T-4	2 Td	Tetrahedron
SP-4	1 D4h	Square planar
	**Coordination number 5**	
JTBPY-5	5 D3h	Johnson trigonal bipyramid J12
SPY-5	4 C4v	Spherical square pyramid
TBPY-5	3 D3h	Trigonal bipyramid
vOC-5	2 C4v	Vacant octahedron
PP-5	1 D5h	Pentagon

Ag1

Structure [ML4]	vTBPY-4	SS-4	**T-4**	SP-4
	8.785,	8.122,	**7.568**,	26.350

Ag2

Structure [ML5]	JTBPY-5	SPY-5	TBPY-5	vOC-5	**PP-5**
	17.865,	13.431,	15.893,	14.601,	**9.200**

Table S11. Classical hydrogen bonds [Å,º] for {[$Ag_2Am4Motaz_3$]$(NO_3)_2 \cdot H_2O$}$_n$ (**7**·H_2O**)**

D-H···A*	dD-H	dH···A	dD···A	<DHA
N15-H15A...O2[3]	0.88	2.26	3.035(6)	146.9
N15-H15B...O13[4]	0.88	2.15	2.971(6)	154.8
N25-H25A...O1[5]	0.88	2.24	2.959(6)	138.3
N25-H25B...O13	0.88	2.11	2.966(6)	165.7
N25-H25B...O12	0.88	2.58	3.175(7)	125.6
N35-H35B...O21[6]	0.88	2.12	2.964(9)	161.1
O4-H4A...O11	0.94	2.29	3.164(9)	154.4
O4-H4B...O13[1]	0.95	2.08	3.010(9)	163.4

[1] x,y+1,z; [2] x,y-1,z; [3]-x+1/2,-y-1/2,-z; [4] x,-y,z-1/2, -z; [5] -x+1/2,y-1/2,-z+1/2; [6] -x,-y+2,-z

*N15, N25 or N35: NH_2 group

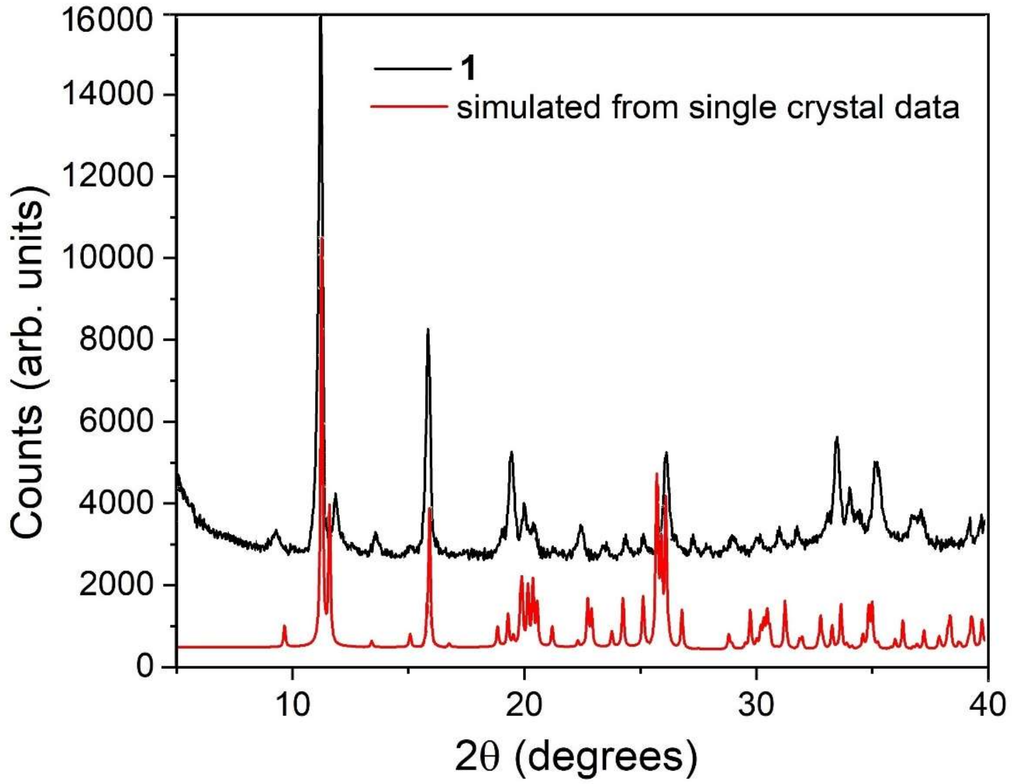

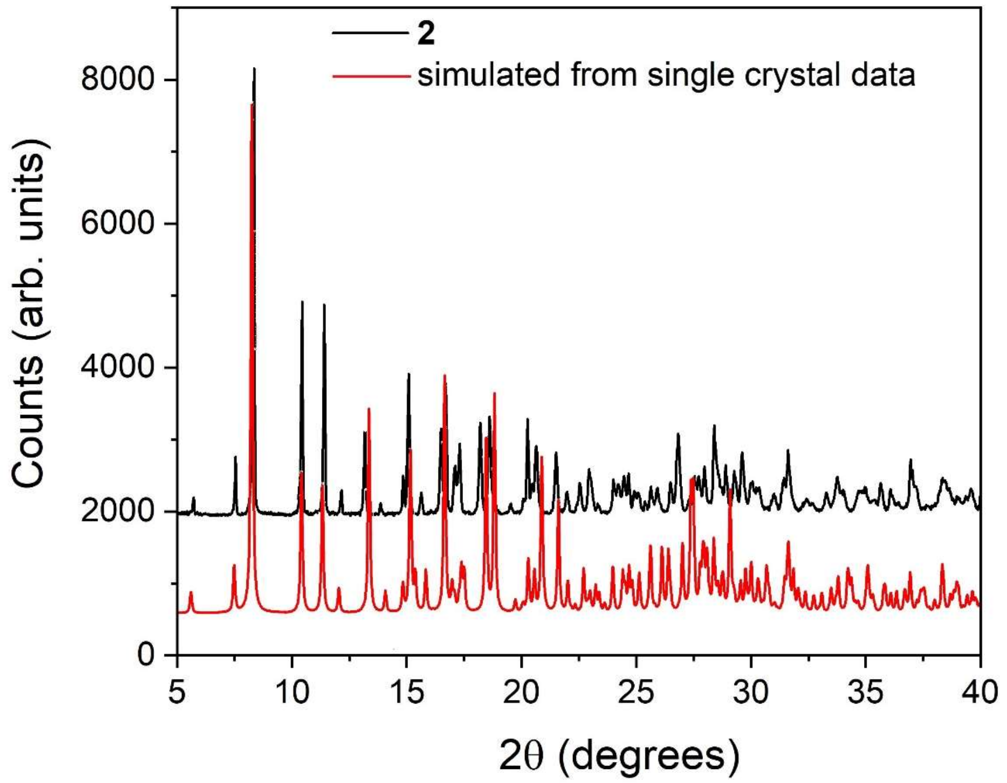

Figure S5. Comparative powder X-ray diffractograms for: up) **1** (blue) and the simulation from single X-ray diffraction data (red). Bottom) **2** (blue) and the simulation from single X-ray diffraction data (red).

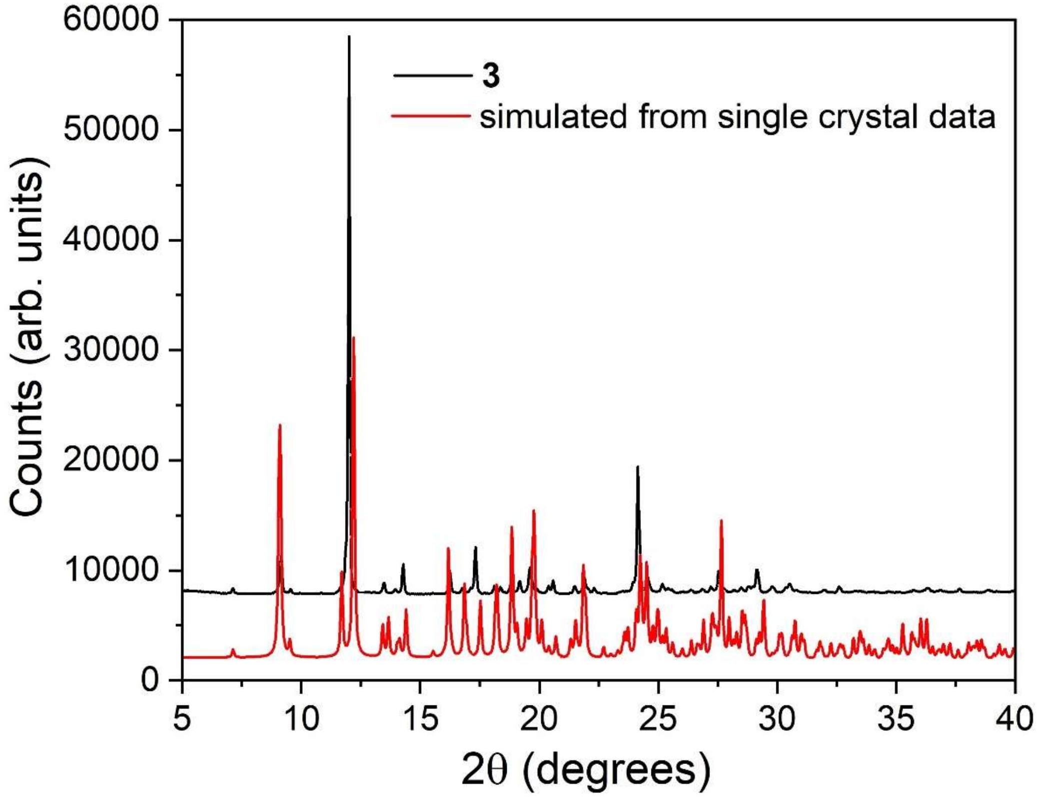

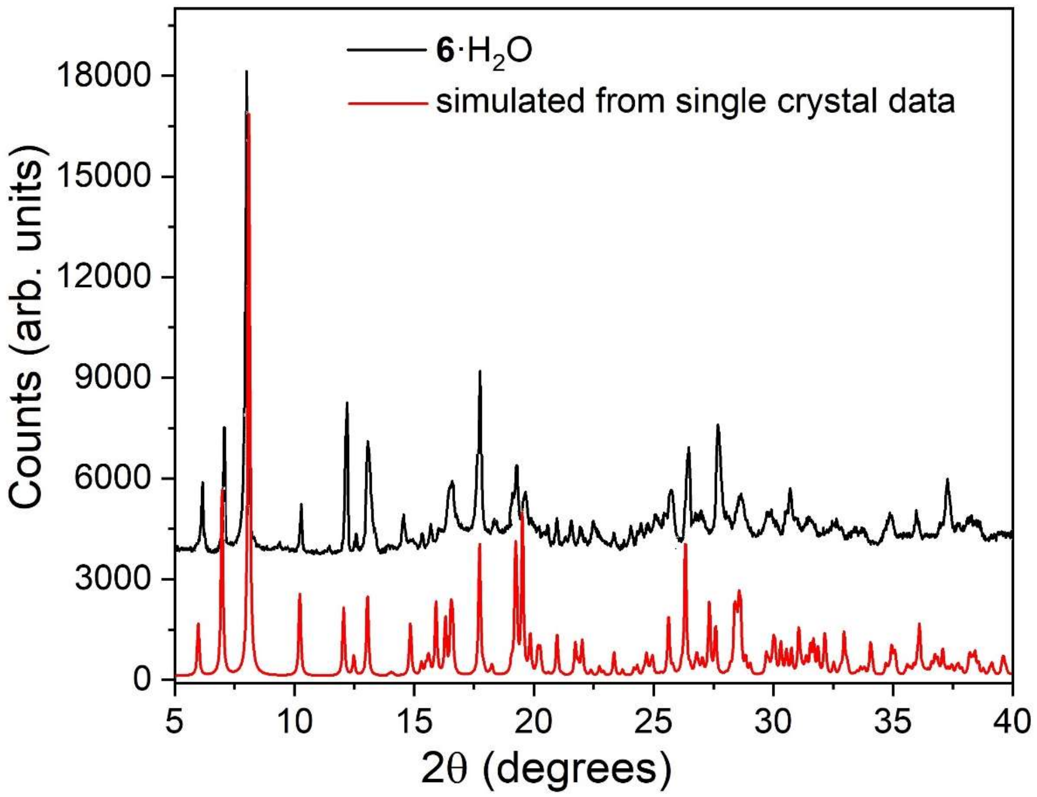

Figure S6. Comparative powder X-ray diffractograms for: up) **3** (blue) and the simulation from single X-ray diffraction data (red). Bottom) **6**·H_2O (blue) and the simulation from single X-ray diffraction data (red).

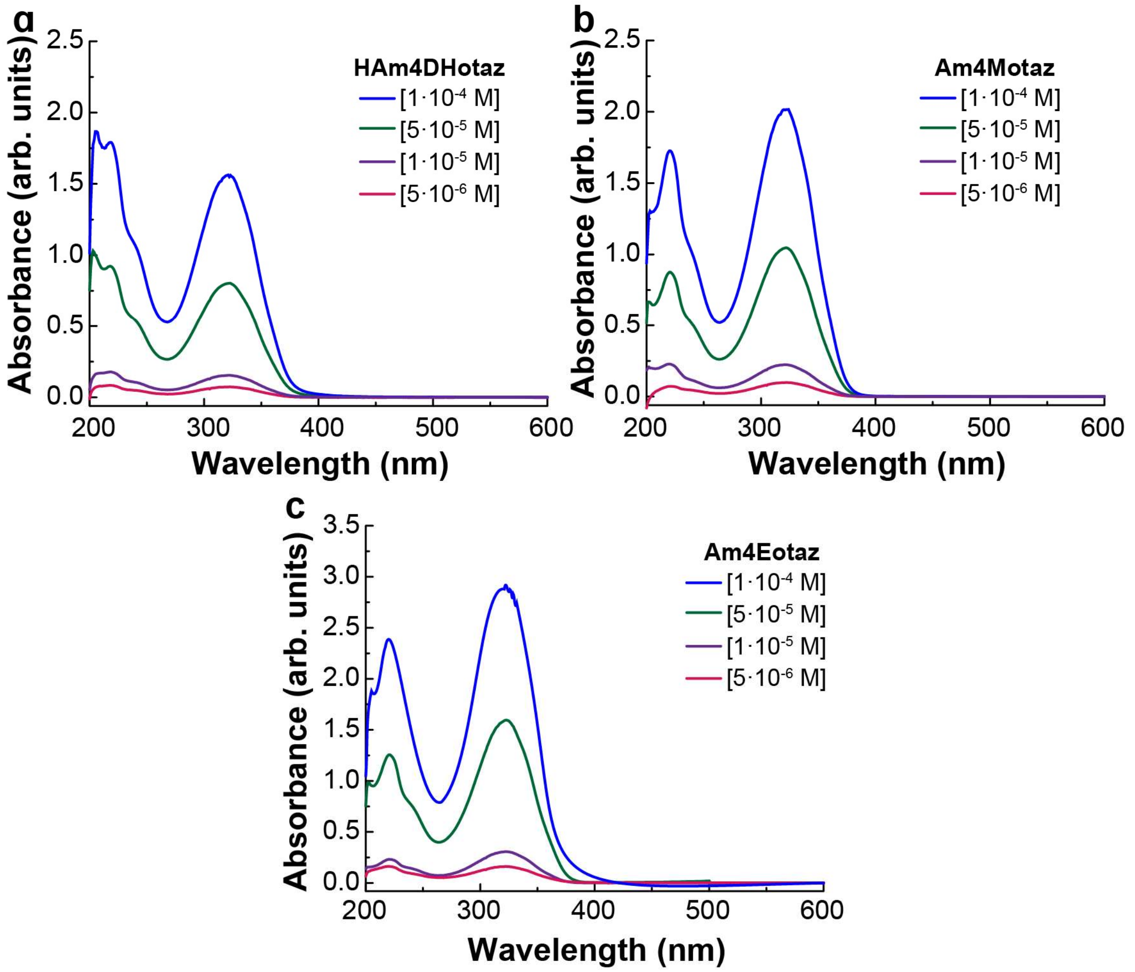

Figure S7. UV–Vis spectra of the ligands a) HAm4DHotaz, b) Am4Motaz, and c) Am4Eotaz in MeOH at different concentrations, recorded at room temperature.

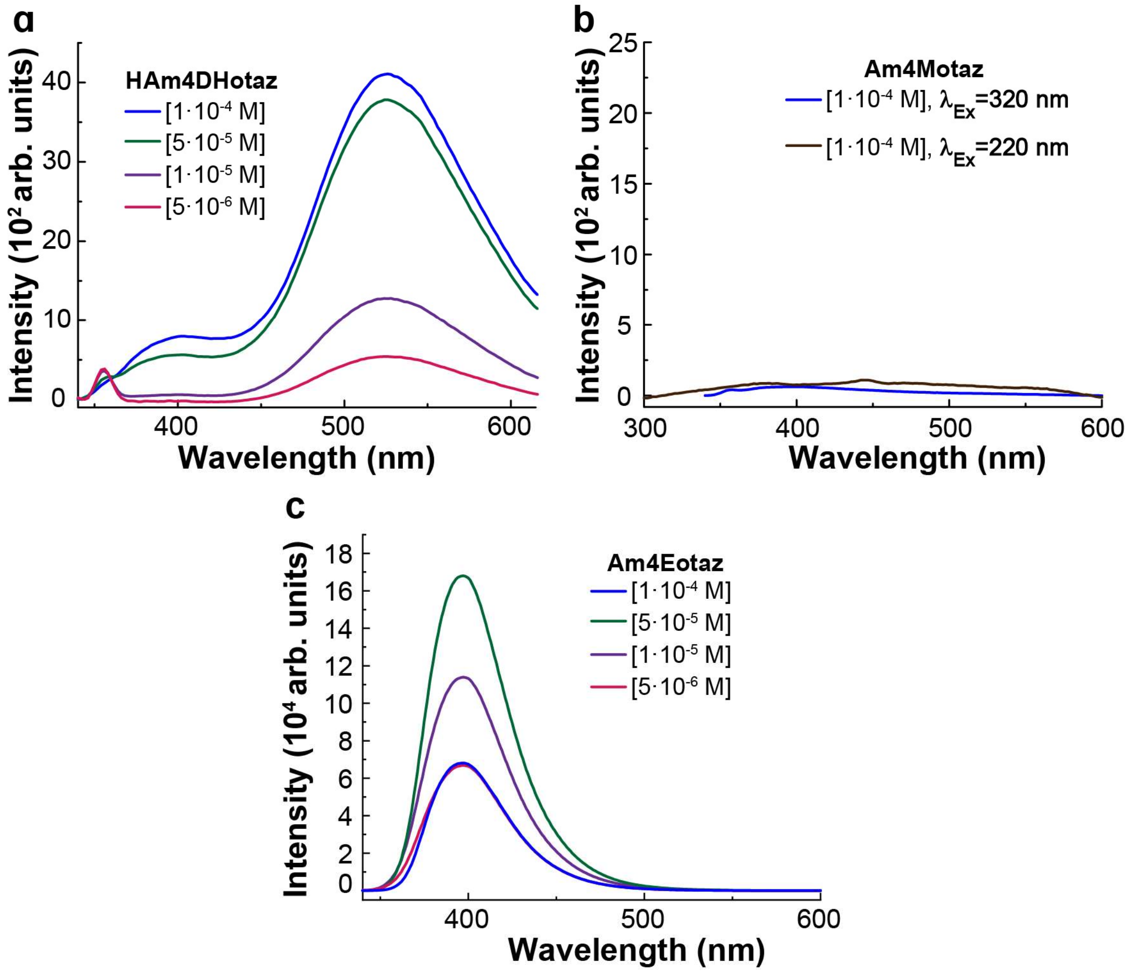

Figure S8. Emission spectra in MeOH recorded at room temperature for a) HAm4DHotaz at different concentrations with λ_{ex} = 320 nm; b) Am4Motaz at a concentration of $1{\cdot}10^{-4}$ M and at different excitation wavelengths (λ_{ex} = 220 nm and λ_{ex} = 320 nm); and c) Am4Eotaz at different concentrations with λ_{ex} = 320 nm.
